# Beyond BMI: A Systematic Review and Meta-Analysis of mHealth Interventions for Pediatric Obesity Management

**DOI:** 10.3390/nu18101511

**Published:** 2026-05-09

**Authors:** Ema Burlacu, Samuel-Andrei Dunăreanu, Cristina Oana Mărginean

**Affiliations:** 1 Doctoral School, “George Emil Palade” University of Medicine, Pharmacy, Science and Technology of Targu Mures, Georghe Marinescu Street No 38, 540136 Mures, Romania; morariuemma@yahoo.com; 2 Clinic of Pediatric Cardiology, County Emergency Hospital Targu Mures, Gheorghe Marinescu Street No 50, 540136 Mures, Romania; 3 Department of Pediatrics 1, “George Emil Palade” University of Medicine, Pharmacy, Science and Technology of Targu Mures, Georghe Marinescu Street No 38, 540136 Mures, Romania

**Keywords:** pediatric obesity, mHealth, zBMI reduction, randomized controlled trial, medical nutrition therapy, hybrid intervention

## Abstract

**Background:** Pediatric obesity (PO) is a chronic disease requiring multidisciplinary management. Recent clinical guidelines emphasize the need for accessible, patient-centered solutions, positioning mHealth interventions as vital “clinical extenders” in modern practice. **Objective:** This systematic review and meta-analysis evaluate the efficacy and evolution of mHealth interventions for PO management between 2020 and 2026. **Methods:** A systematic search of electronic databases identified randomized controlled trials (RCTs) investigating mHealth for PO. Quality was assessed using the Cochrane RoB 2 tool, and a meta-analysis was performed on a subset of studies reporting zBMI data. **Results:** Twenty-three RCTs met the inclusion criteria, of which six were included in the quantitative synthesis. The meta-analysis demonstrated a statistically significant reduction in BMI z-score (MD = −0.20; 95% CI: −0.36 to −0.04; *p* = 0.02), with moderate heterogeneity (*I*^2^ = 60%; Q = 13.5, *p* = 0.019). Beyond anthropometric outcomes, mHealth interventions consistently improved behavioral parameters, including dietary quality and sedentary time. However, engagement declined over time in standalone digital interventions (“mHealth fade-out”), whereas hybrid models integrating human support demonstrated improved retention and sustained effects. Anthropometric and behavioral outcomes showed partially divergent trajectories, with behavioral improvements often preceding measurable changes in BMI, and data on body composition were rarely reported, limiting a more precise understanding of changes in adiposity beyond BMI. **Conclusions:** mHealth is an effective catalyst for obesity management when integrated into a multidisciplinary framework. Future protocols must prioritize developmental tailoring—targeting parental empowerment in early childhood and encouraging adolescent autonomy—to ensure sustained engagement and a clinical focus that looks beyond BMI.

## 1. Introduction

Pediatric obesity (PO) represents one of the most significant challenges to global public health in the 21st century, necessitating an intensified and multifaceted response from the medical community [1,2,3]. Far from being a simple condition of excessive adipose tissue, obesity is now recognized as a complex, chronic disease with the potential to impair multi-systemic health across the entire lifespan [4]. A recent joint position statement by the European Association for the Study of Obesity (EASO) and the European Federation of the Associations of Dietitians (EFAD) emphasizes that obesity must be formally recognized as a chronic disease to prioritize health policies and long-term treatment strategies. This authority highlights that management should be oriented towards patient-centered health outcomes and quality of life, moving beyond a weight-centric approach to mitigate weight-related stigma [5]. At the same time, the prevalence of pediatric obesity has reached alarming proportions worldwide. According to recent World Health Organization (WHO) data, approximately 37 million children under the age of five and 390 million children and adolescents aged 5–19 were classified as overweight or obese [6,7]. Longitudinal analyses indicate that this trajectory is worsening; for instance, youth obesity in the United States rose from 17.7% in 2011 to 21.5% by 2020 [8]. Projections remain dire, with a forecasted 60% increase in PO rates by 2030 [9]. This crisis was further exacerbated by the COVID-19 pandemic, where sedentary behaviors and dietary shifts led to a documented “spike” in severe obesity cases [10,11]. The clinical and economic ramifications of PO are profound. Adolescent obesity is intrinsically linked to an increased risk of type 2 diabetes and cardiovascular disorders in adulthood [12]. Furthermore, a high Body Mass Index (BMI) during adolescence correlates with non-alcoholic fatty liver disease (NAFLD), polycystic ovary syndrome (PCOS), and various malignancies [13]. In addition to physical health complications, pediatric obesity is strongly associated with psychosocial distress, including internalized weight bias and low self-esteem, contributing to a substantial burden on global healthcare systems [14]. Given these multifaceted consequences, effective and sustainable treatment strategies are essential. Traditional face-to-face, multicomponent behavioral interventions are considered the gold standard, yet they often struggle with sustainability and accessibility [15,16]. In this context, mobile health (mHealth) technologies have emerged as a transformative solution. mHealth platforms—encompassing smartphone applications, SMS support, and telehealth—offer a cost-effective alternative to home-visiting programs [17]. These digital tools effectively overcome traditional barriers such as geographical isolation and busy family schedules [18,19].

Pediatric health is increasingly recognized as a multidimensional construct, with clinical outcomes extending beyond disease-specific markers to include broader domains such as quality of life, treatment response, and long-term health trajectories, as observed across diverse pediatric conditions [20,21]. The clinical utility of digital health has been recently consolidated by large-scale systematic reviews. Notably, Azevedo et al. demonstrated through meta-analysis that e-health interventions are associated with a significant reduction in BMI z-scores [22]. This underscores that digital platforms are effective channels for treatment, aligning with the EASO (European Association for the Study of Obesity) and EFAD (European Federation of the Associations of Dietitians) recommendations that such interventions should be adequately resourced and implemented in various formats—including digital or online delivery—to enhance accessibility [23]. However, there remains a critical need to understand which “active ingredients” drive long-term sustainability, as technological shifts often lead to decreased adherence over time [22,24]. The critical importance of digital health was solidified during the COVID-19 pandemic, when mHealth became the primary vehicle for maintaining continuity of care [25]. Despite the growing body of literature on digital health interventions for pediatric obesity, several important gaps remain. Existing systematic reviews and meta-analyses have predominantly focused on anthropometric outcomes, particularly BMI and BMI z-score, often neglecting behavioral parameters such as dietary quality, physical activity, sedentary behavior, and intervention engagement [26]. Furthermore, much of the available evidence is based on studies conducted before or during the early phases of the COVID-19 pandemic, limiting its applicability to the rapidly evolving post-pandemic digital health landscape. In addition, patterns of engagement and sustainability of effects appear to vary across mHealth interventions, particularly over longer follow-up periods.

**Figure 1:**
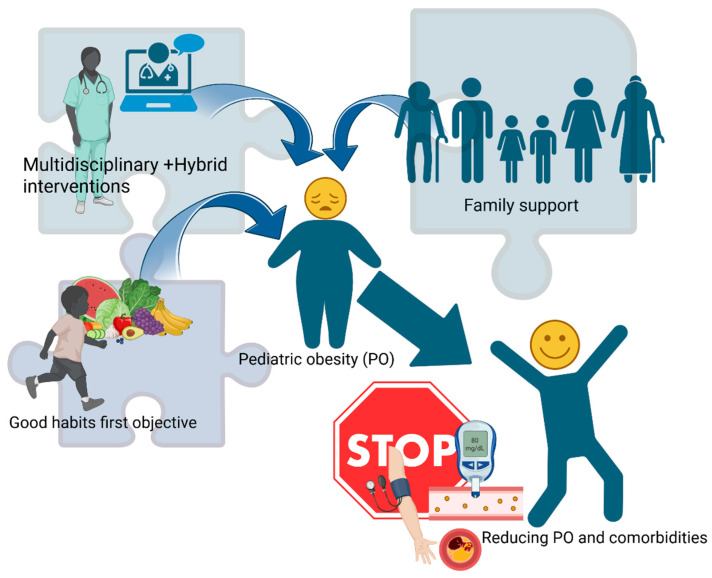
Conceptual framework of mHealth interventions in pediatric obesity management. The figure illustrates the interplay between multidisciplinary hybrid interventions, family support, and behavioral changes, contributing to the development of healthy habits and the reduction in obesity-related comorbidities.

The conceptual framework of mHealth interventions in pediatric obesity, including the interplay between multidisciplinary and hybrid care, family support, and behavioral changes leading to improved outcomes, is illustrated in Figure 1.

To address these gaps, this systematic review and meta-analysis aimed to quantify the pooled effect of mHealth interventions on BMI z-score and to synthesize outcomes beyond BMI, including behavioral parameters and engagement-related findings in children and adolescents with overweight and obesity.

## 2. Materials and Methods

### 2.1. Search Strategy and Information Sources

To ensure comprehensive and unbiased coverage of the literature, a multi-database search strategy was implemented. The protocol for this systematic review was not prospectively registered in an external database, such as PROSPERO (University of York in England); however, the study was conducted in accordance with the PRISMA 2020 ((Melbourne, Australia), Preferred Reporting Items for Systematic Reviews and Meta-Analyses) statement to ensure methodological transparency and quality. The completed PRISMA 2020 checklist is provided as Supplementary Material S1. The systematic search was conducted across five major electronic databases: MEDLINE (via PubMed), Web of Science (Core Collection), and Scopus served as the primary sources for the peer-reviewed medical literature. Additionally, to maximize retrieval sensitivity and capture emerging evidence not yet fully indexed in traditional databases, the search was extended to Dimensions.ai and Lens.org. These multidisciplinary platforms aggregate data from Crossref, Microsoft Academic, and various digital repositories, ensuring that recent technological trials were captured. Despite the rigorous methodological frameworks proposed in recent years for assessing mHealth apps in pediatric obesity—such as the comprehensive multi-database approach outlined by Rashid et al. (2020) [27]—there remains a notable gap between protocol intentions and published longitudinal evidence. This underscores the urgency of our synthesis in providing an up-to-date evaluation of the clinical effects of these technologies.

The search covered the period from 1 January 2020 to 29 January 2026 across all primary databases (PubMed, Web of Science, Dimensions.ai, and Lens.org). To ensure the inclusion of the most recently indexed literature prior to manuscript finalization, a targeted update was performed exclusively in Scopus on 28 March 2026. This strategic update focused on Scopus due to its comprehensive indexing of the latest randomized controlled trials in digital health. We deliberately restricted the search to this timeframe to capture the latest advances in mobile health technologies relevant to pediatric obesity management. This period coincides with the global COVID-19 pandemic, a catalyst for the rapid expansion of digital health solutions. By focusing on this interval, we aim to provide an updated synthesis of evidence-based mHealth interventions, highlighting both technological innovations and their clinical efficacy under real-world conditions.

The search strategy utilized a combination of Medical Subject Headings (MeSH/Keywords) and free-text terms relevant to three key domains: Population, e.g., ‘P(a)ediatric Obesity’, ‘Childhood Overweight’, ‘Adolescent’; Intervention, e.g., ‘mHealth’, ‘Smartphone App’, ‘Telemedicine’, ‘eHealth’; Study Design, e.g., ‘Randomized Controlled Trial’, ‘Clinical Trial’.

To ensure the inclusion of the most recent data, a manual hand-search of key journals and a snowballing process (screening reference lists of eligible studies) were also performed. This rigorous update led to the identification of recent pivotal trials [28,29] that were not yet indexed in the initial electronic search. The search strategy is summarized in Table 1, while the complete database-specific search strings and search histories are provided in Supplementary Material S2.

In preparing this manuscript, generative AI tools were used only to improve language and grammar. All scientific content, data extraction, and interpretation of the results were performed solely by the authors.

### 2.2. Study Selection, Data Extraction, and Eligibility Criteria

The screening process was conducted independently by two reviewers using the Rayyan platform (AI-Powered Systematic Review Management Platform version, Qatar Computing Research Institute, Cambridge, MA, USA/Doha, Qatar) [30]. Data extraction was performed by one reviewer using a standardized data extraction form and subsequently checked for accuracy and completeness by a second reviewer. Extracted variables included study characteristics, participant demographics, intervention details, and outcome measures. Any discrepancies were resolved through discussion and consensus.

Studies were included if they met the following criteria:

1. Peer-reviewed randomized controlled trials;
2. Pediatric population (0–18 years);
3. mHealth or eHealth interventions for obesity management;
4. Reporting anthropometric outcomes (e.g., BMI z-score, BMI SDS, body composition) with follow-up assessment.

Studies were excluded if they were reviews, meta-analyses, observational studies, editorials, protocols, conference abstracts without full text, non-English publications, or duplicate records. Studies involving adult populations, animal models, or laboratory-based designs were also excluded.

The full search history, including the specific query syntax and the retrieved records, was archived and is available as Supplementary Material S2 to ensure transparency and reproducibility.

### 2.3. Statistical Analysis and Meta-Analysis Methods

To evaluate the effectiveness of mHealth interventions on pediatric BMI z-score (zBMI), a meta-analysis was performed using the generic inverse variance method. This approach was selected to accommodate the heterogeneity in outcome reporting across the included randomized controlled trials (RCTs), allowing for the synthesis of effect estimates provided in diverse formats (e.g., mean differences, confidence intervals, or change scores). Effect sizes were expressed as mean differences (MDs) between intervention and control groups. Standard errors were calculated from reported standard deviations or derived from 95% confidence intervals (CIs); when variance data were not directly reported, they were estimated using available summary statistics. A random-effects model was applied using the restricted maximum likelihood (REML) estimator to account for between-study variability. For pooled estimates, 95% CIs were calculated using the Hartung–Knapp adjustment, providing a more conservative and robust estimate suitable for a limited number of studies (k < 10). Statistical heterogeneity was assessed using Cochran’s Q test (significance level at *p* < 0.05) and the *I*^2^ statistic, with values around 50–60% considered as moderate heterogeneity. To estimate the range of true effects in future clinical settings, 95% prediction intervals were calculated. Publication bias was evaluated through a multi-step approach, including funnel plot inspection, the Duval and Tweedie trim-and-fill analysis to estimate the impact of potentially missing studies, and the Rosenthal fail-safe N to assess the stability of the findings against unpublished null results. Sensitivity analysis was systematically conducted by excluding studies requiring substantial data approximation to ensure the robustness of the final synthesis.

Potential sources of heterogeneity, including participant age, intervention type (standalone vs. hybrid), and intervention duration, were explored qualitatively due to the limited number of included studies.

## 3. Results

The systematic search conducted across MEDLINE (via PubMed), Web of Science, Scopus, Dimensions.ai, and Lens.org yielded a total of 208 records. After the removal of 108 duplicates, 100 unique citations were screened by title and abstract. A total of 78 records were excluded during the initial phase as they did not meet the predefined inclusion criteria (e.g., non-RCT designs, adult populations, or lack of mHealth components). During the abstract-only and full-text selection, studies were excluded primarily due to: Methodological limitations: lack of a randomized control group; Population criteria: studies focusing on normal-weight children without anthropometric follow-up; Outcome reporting: absence of anthropometric measurements. Subsequently, 22 articles were sought for retrieval and assessed for eligibility. One study represented a secondary analysis and was consequently excluded from the analytic sample.

The systematic search across electronic databases initially identified 21 eligible trials. To ensure a comprehensive synthesis, we performed an additional manual search strategy, including citation tracking (snowballing) and the screening of reference lists from relevant systematic reviews and included studies. This process yielded 2 additional studies that met the inclusion criteria [28,29].

**Figure 2:**
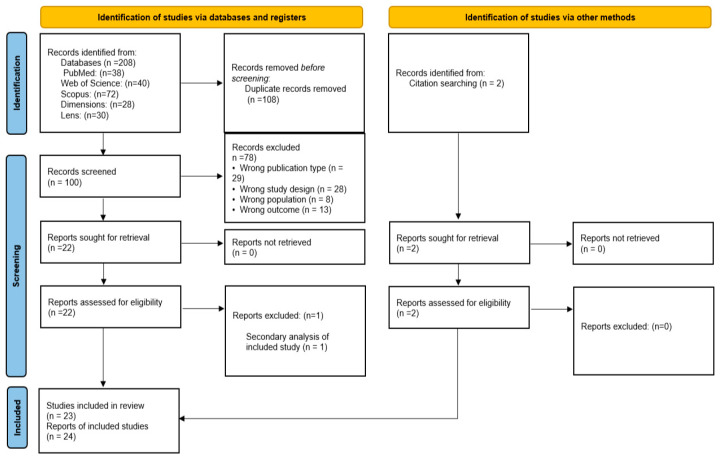
PRISMA flowchart for assessment of eligible studies [34].

Ultimately, 23 Randomized Controlled Trials met all criteria and were included in the final qualitative synthesis. The complete selection process is documented in the PRISMA Flow Diagram (Figure 1) [31]. The final inclusion of 23 unique RCTs corresponds to 24 reports; one study [32] was supplemented with data from a secondary analysis [33] to ensure comprehensive data extraction regarding parental engagement (Figure 2).

The methodological quality of the 23 included randomized controlled trials was assessed using the Cochrane Risk of Bias 2 (2019 RoB 2 version) tool [35]. Each study was evaluated across five distinct domains: (1) bias arising from the randomization process; (2) bias due to deviations from intended interventions; (3) bias due to missing outcome data; (4) bias in the measurement of the outcome; (5) bias in the selection of the reported result.

Based on the signaling questions provided by the RoB 2 framework, each domain was rated as having a ‘Low risk of bias’, ‘Some concerns’, or a ‘High risk of bias’. The overall risk of bias for each study was determined by the highest risk identified in any of the individual domains. Discrepancies between the two independent reviewers during the assessment process were resolved through consensus. The detailed quality assessment for all included trials is summarized in Table 2.

Overall, 4 studies (17%) were rated as Low Risk, 14 (61%) as having Some Concerns, and 5 (22%) as High Risk. High attrition rates (>20%) and the use of parent-reported measurements were the most common sources of potential bias.

### 3.1. Characteristics of Included Studies

The 23 included randomized controlled trials (RCTs) present significant heterogeneity in terms of study objectives, population demographics, and technological delivery. While the majority of the research focused on the treatment of established pediatric obesity (18 studies; 78.2%), five trials specifically targeted early prevention or management of obesity risk in infants and preschoolers [39,45,46,47,54].

The inclusion criteria generally aligned with international standards for pediatric weight status. Most treatment-oriented studies included children or adolescents with a BMI ≥85th percentile or ≥95th percentile for age and sex [32,38,41,48,51,55], while some focused on severe obesity (BMI z-score > 2 SD) or high-risk populations with comorbidities [37,49,50]. Preventive interventions primarily targeted early-life populations through parental involvement, particularly in infants and preschool children [44,45,46,47,54].

Sample sizes ranged from small feasibility and pilot trials to large-scale, school-based clusters. Overall, 11 of the 23 studies (47.8%) included more than 100 participants.

Geographically, the evidence base is distributed globally across 14 countries. Sweden [28,36,47,53], Australia [39,51,54], and the USA [41,46,49] contributed the largest number of trials. Significant contributions also came from Italy [38,48] and, Iran [43,44].

The classification by age group revealed three main clusters: infancy and preschool (0–5 years)—4 studies [45,46,47,54]; school-age children (6–12 years)—11 studies [28,29,36,37,39,42,43,44,48,49,51]; adolescents (13–18 years)—8 studies [32,38,40,41,50,52,55].

Intervention duration varied substantially across studies, ranging from short-term interventions of 8–12 weeks to long-term follow-up periods of up to 3 years, with most interventions lasting between 3 and 12 months.

Table 3 summarizes study characteristics, including country, population, intervention type, comparison group, duration, and study design.

### 3.2. Primary Outcomes: Anthropometric and Behavioral Efficacy

Nineteen of the twenty-three studies (82.6%) used a smartphone application or mobile messaging as the primary delivery vehicle [32,36,37,38,41,42,43,44,45,46,47,48,50,52,53,55].

In terms of anthropometric changes, 12 out of 23 trials (52.1%) demonstrated a statistically significant difference in BMI or zBMI that favored the intervention group or showed significant within-group reductions [28,29,36,37,38,42,43,45,49,51,53,54].

The largest long-term reduction was reported by Hagman et al. [53], where a “digi-physical” model achieved a zBMI change of −0.29 compared to −0.12 in standard care, doubling the remission rate. Significant standardized BMI reductions were also observed by Thorén et al. [28] (−0.27 zBMI) in a hybrid web-portal model and Johansson et al. [36] (−0.23 BMI SDS). In early childhood, Wen et al. [54] reported a significant mean BMI reduction (diff: −0.30), with even stronger effects noted in low-income families.

Metabolic outcomes were reported in several studies. Salahshoornezhad et al. [43] and Audi et al. [55] both reported significant improvements in metabolic markers; specifically, Audi et al. demonstrated that while zBMI decreased in both groups, the app-using cohort achieved superior improvements in insulin resistance (HOMA-IR) and HDL-cholesterol.

Behavioral outcomes, including dietary intake and screen time, were also reported. Several studies reported behavioral improvements without significant BMI or zBMI changes. Lee et al. [46] observed a significant increase in fruit (+0.89 servings) and vegetable intake (+0.60 servings) alongside a 33.8 min reduction in daily screen time in toddlers after only 8 weeks. Similar dietary improvements were noted by Alexandrou et al. [47] regarding sweet drink consumption and by Memarian et al. [44] through a gamified inhibitory control app that reduced sweet food intake despite no immediate zBMI change.

Retention rates varied across studies. High retention was maintained in studies involving early childhood caregivers, such as Lee et al. [46] (93%) and Alexandrou et al. [47] (93%). Conversely, adolescent cohorts faced higher attrition, with Mateo-Orcajada et al. [50] documenting a critical app abandonment rate of over 50% by week 6. In Umano et al. [48] study, dropout was lower in the intervention group than in the control group, and they found that app usage significantly reduced clinical dropout (40% vs. 72% in control), while Foissac et al. [52] achieved 92% retention by fostering adolescent autonomy through remote monitoring.

Regarding specific populations, Davis et al. [49] reported significant zBMI differences in rural cohorts at 20 months. For low-income families, Lee et al. [46] and Wen et al. [54] observed significant improvements in fruit and vegetable intake and reduced television-side eating. Long-term data from Karssen et al. [45] showed that the zBMI reduction achieved at 6 months was no longer present at the 12-month follow-up.

The detailed clinical effects, including changes in zBMI, metabolic parameters, and behavioral metrics, alongside study retention and digital engagement rates, are systematically synthesized in Table 4.

### 3.3. Characteristics of Included Interventions

Parental involvement was included in preschool and school-age trials [45,46,47,48,54]. Gamification, including point systems, rewards, and interactive challenges, was used in several studies [32,43,44] (Table 5).

Hybrid models, combining mHealth tools with traditional clinical care, physical visits, or human coaching, were employed in 52% of the included studies (12 out of 23) [28,36,37,38,40,46,48,49,51,52,53,54]. In these “blended care” models, Umano et al. [48] reported a significantly lower clinical dropout rate in the intervention group (40%) compared to the control group (72%).

Several trials conducted or extended during the COVID-19 pandemic reported attrition rates ranging from 39% to 47% and required remote measurements, such as Tsai et al., Vidmar et al. and Wen et al. [29,41,54] (Table 5).

Notably, only 21.7% (*n* = 5/23) of studies reported direct body composition outcomes, highlighting a critical gap in the current evidence base, where BMI remains the predominant—yet insufficient—marker of intervention efficacy [29,40,42,50,55].

Body composition outcomes were inconsistently reported across the included studies. Only a minority of trials assessed direct parameters such as fat mass, body fat percentage, or lean mass. Specifically, Stasinaki et al. [40] and Audi et al. [55] used bioelectrical impedance analysis, while Tsai et al. estimated body fat percentage using skinfold measurements. Mateo-Orcajada et al. [50] also assessed body composition using anthropometric estimation methods; however, no significant improvements were observed. In contrast, most studies relied exclusively on anthropometric indicators such as BMI, BMI z-score, or surrogate measures (e.g., waist circumference or waist-to-height ratio), without reporting detailed body composition outcomes. Due to differences in participant age, intervention design, and outcome reporting, only six trials provided sufficiently comparable anthropometric data for quantitative synthesis. The remaining studies were included in the qualitative analysis.

### 3.4. Meta-Analysis Results: Impact of mHealth on zBMI

**Figure 3:**
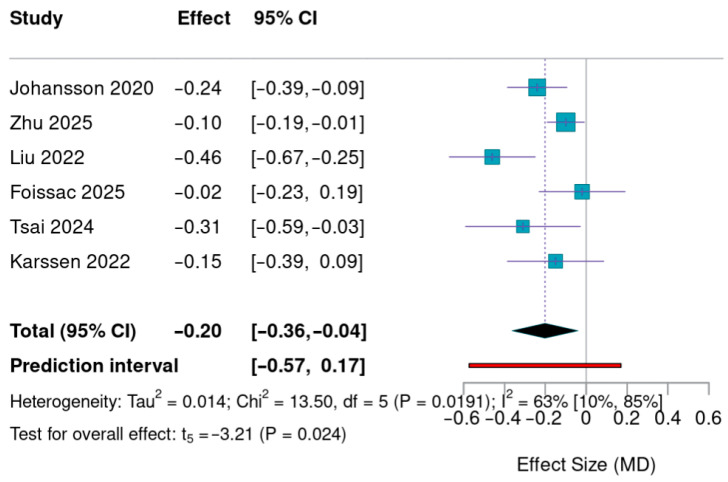
Forest plot illustrating mean differences (MDs) in ZBMI reduction between the intervention and control groups (95% CI) [29,36,42,45,51,52].

A total of six randomized controlled trials (*n* = 6) were included in the quantitative synthesis, which represents a limited evidence base for meta-analytic estimation [29,36,42,45,51,52]. The pooled analysis demonstrated a statistically significant reduction in BMI z-score in the mHealth intervention groups compared to controls (MD = −0.20; 95% CI: −0.36 to −0.04; *p* = 0.023) (Figure 3).

Moderate heterogeneity was observed (*I*^2^ = 60%), indicating variability in effect estimates across studies. Sensitivity analyses confirmed the direction of the effect; however, statistical significance was affected when studies requiring data approximation were excluded (Figure 3).

Publication bias analyses suggested potential asymmetry, with the trim-and-fill method identifying two potentially missing studies. After adjustment, the pooled effect size was attenuated and no longer statistically significant (adjusted MD = −0.13). The Rosenthal fail-safe N of 1 further indicates that the results may be sensitive to the inclusion of additional null-effect studies (Figure 3).

Taken together, these findings should be interpreted with caution due to the small number of included studies, the presence of moderate heterogeneity, and the potential impact of publication bias. Regarding publication bias, the trim-and-fill analysis identified two potentially missing studies on the right side of the funnel plot. After adjusting for these studies, the pooled effect size was reduced and became non-significant (adjusted MD = −0.13). Furthermore, the Rosenthal fail-safe N was calculated at 1, suggesting that the addition of a single null-result study could potentially bring the *p*-value above the significance threshold (Figure 3).

Individual study estimates are represented by squares (size proportional to study weight), with horizontal lines indicating 95% confidence intervals (CIs). The pooled effect size is depicted as a black diamond. The vertical solid line (MD = 0) represents the null effect. The red horizontal line illustrates the prediction interval (−0.57 to 0.17), indicating the expected range of effects in future settings. MD: Mean Difference.

Detailed statistical metrics and data approximation procedures are provided in Supplementary Material S3.

### 3.5. Qualitative Synthesis of Research Gaps and Clinical Observations

Table 6 summarizes study-specific findings, reported research gaps, and key observations extracted from the included trials. The qualitative synthesis includes all 23 studies and complements the quantitative analysis by presenting non-standardized outcomes, including behavioral, engagement-related, and implementation-related findings.

More detailed versions of Table 3 and Table 4, and 6, including extended study-level data, are available in Supplementary Material S4.

## 4. Discussion

### 4.1. Characteristics of Included Studies: Diversity as a Methodological Challenge

The findings of this systematic review reflect the rapid evolution and inherent complexity of the mHealth landscape in pediatric weight management. While the results are promising, with over 55% of the included trials reporting significant anthropometric improvements, the marked disparity in study designs and intervention protocols aligns with recent evidence highlighting the extreme heterogeneity of digital health research [18,22,56].

The methodological quality of the included studies should also be considered when interpreting the findings. The predominance of studies categorized under ‘Some Concerns’ (61%) primarily reflects the inherent challenges of mHealth behavioral trials, where double-blinding is often pragmatically impossible. The notable incidence of ‘High Risk’ in the missing outcome data domain (D3), identified in 5 trials, is closely linked to the ‘mHealth fade-out’ effect, where digital disengagement led to significant attrition, particularly in adolescent cohorts [41,50]. Furthermore, the reliance on parent-reported metrics in several trials (D4) introduces a risk of social desirability bias, potentially overestimating treatment efficacy [39,45]. Consequently, while the emerging evidence is promising, the findings from high-attrition studies should be interpreted with caution.

As noted in previous meta-analyses, this lack of methodological standardization across mHealth RCTs continues to be a primary barrier to performing robust pooled analyses, with heterogeneity often exceeding 70–90% [22,57]. Our synthesis suggests that mHealth efficacy is deeply contingent upon the developmental stage of the child and the degree of parental involvement [58]. This observation is consistent with recent evidence suggesting that mHealth interventions are generally more effective for treatment rather than prevention, often yielding a standardized mean difference (SMD) in BMI z-score ranging from −0.13 to −0.35 [18,59,60]. Furthermore, the success of the hybrid models identified in 50% of our included trials is consistent with previous evidence suggesting that teleinterventions are most effective when they incorporate structured professional interaction and family engagement [57,59]. Despite these benefits, the “mHealth fade-out” effect remains a critical challenge. While short-term improvements (4–6 months) in BMI are frequently significant, these effects often diminish during longer-term follow-ups (12 months), as seen in Karssen et al. [45]. However, the inclusion of the 3-year pragmatic trial by Hagman et al. provides longer-term evidence suggesting that a “digi-physical” model can sustain weight loss and double remission rates over 36 months, provided that parental empowerment and frequent objective monitoring are maintained [45]. The sample sizes ranged from small pilot feasibility trials [36,39] to large-scale, school-based clusters [42], suggesting that mHealth interventions may be applicable across both clinical and public health instruments. As noted by Mihrshahi et al. [61], the heterogeneity in delivery timing and setting (home vs. school vs. clinic) often restricts the comparison of long-term outcomes. This setting-specific impact was also observed by Rybak et al. [62] in the THRIVE 2.0 trial, where caregiver engagement was found to be highly dependent on the baseline socioeconomic context of the families [62,63].

A key source of heterogeneity lies in the targeted developmental stages. We observed that interventions for infants and toddlers successfully utilized parents as proxy agents of change, focusing on “responsive feeding” [45,64]. Lee et al. [46] and Wen et al. [54] reinforce this by showing that even asynchronous video guidance or nurse-led telehealth can significantly improve dietary variety during the first 2000 days of life. This approach was early established by the MINISTOP trial [65], which demonstrated the feasibility of using smartphone technology to target parental behaviors in 4-year-olds [65]. Similarly, the Smart Moms trial utilized mobile-based methods to specifically target mother-child dyads, focusing on reducing sugar-sweetened beverage (SSB) intake in preschoolers as a strategy to address maternal weight status—one of the strongest predictors of early childhood obesity [66]. Similarly, the Norwegian Early Food for Future Health trial (Helle et al.) demonstrated that an eHealth intervention providing monthly video guidance can significantly improve infant eating behaviors, increasing vegetable variety and promoting beneficial mealtime routines, such as family breakfasts and tablet-free meals, during the critical transition to solid foods [67]. However, these demographics face higher attrition rates when the digital “voice” of the app fails to resonate [50,68]. In contrast, adolescent-targeted interventions required designs that respect autonomy and peer-influence [29,49]. As evidenced by Audi et al. [55], these digital tools may offer a potentially advantageous alternative for adolescents reducing the fear of external judgment and providing a “stigma-free” interface for metabolic health monitoring.

The findings of this systematic review reflect the rapid evolution and inherent complexity of the mHealth landscape in pediatric weight management. Finally, while Salahshoornezhad et al. [43] and Audi et al. [55] suggest that mHealth interventions may be associated with improvements in metabolic parameters (e.g., HOMA-IR and HDL-c improvements) even in the absence of dramatic weight loss, translating behavioral shifts into sustained adiposity reduction remains a challenge. The ATLAS trial focused on adolescent boys and similarly demonstrated sustained behavioral shifts, such as reduced screen time, without achieving significant long-term reductions in adiposity [69]. Similarly, Chen et al. demonstrated that being female and reducing sugary beverage intake were significantly related to BMI z-score reduction in a culturally tailored smartphone intervention for Chinese American adolescents [70]. This suggests that while behavioral shifts are achievable across genders, translating them into sustained anthropometric changes remains a universal challenge in adolescent mHealth interventions, and mHealth research should move “beyond BMI” to include broader cardiometabolic and quality-of-life indicators as primary measures of success.

### 4.2. The Nutritional “Dose-Response” and Behavioral Efficacy

A critical factor influencing efficacy appears to be the “dose” of intervention. The USPSTF (U.S. Preventive Services Task Force), an independent panel of experts that formulates evidence-based recommendations for clinical preventive services, recommends ≥26 contact hours for effective treatment [49,71], a threshold rarely met by standalone apps. As highlighted by Davis et al., even in successful telehealth interventions, the actual dose received (15.8 h) often falls short of these international recommendations, potentially limiting long-term anthropometric impact. We observed that studies achieving significant BMI SDS reductions [28,29] were those that integrated digital tools into a broader multidisciplinary framework. This may suggest that in nutritional interventions, the “app” functions as a reinforcement for the “advice,” rather than as a standalone therapist. The high adherence reported by Zhu et al. [51], where families completed 90% of modules, further suggests that combining digital content with human accountability (e.g., weekly coaching calls) may be important for maintaining the necessary “dose” of engagement.

Our analysis reveals a divergence between anthropometric and behavioral outcomes. Consistent with the findings of Bendtsen et al. [72] and Alexandrou et al. [47], mHealth tools often improve diet quality—specifically reducing sugar-sweetened beverage (SSB) intake—even before a significant change in zBMI is detected. This behavioral “re-patterning” is prominently seen in the results of Lee et al. [46], who documented significant increases in fruit and vegetable intake alongside a substantial reduction in screen time (−33.8 min/day) in toddlers after only 8 weeks of video-based intervention. As Rosenbaum [73] argued, these habit-based shifts, such as improved eating rhythms and reduced ultra-processed food consumption, are better predictors of long-term cardiometabolic health [73,74]. As an example, the “SWAP IT” trial further supports the role of parental influence, suggesting that mHealth interventions may improve the nutritional quality of school lunchboxes through digital ‘nudges’ and resources [75]. In line with these findings, Rhodes et al. demonstrated that a short-term (5-week) telephone-based intervention successfully fostered prescribed dietary shifts, such as reduced glycemic load or fat intake, emphasizing the utility of remote counseling in achieving rapid behavioral differentiation [76]. Furthermore, the metabolic benefits observed by Audi et al. [55] and Salahshoornezhad et al. [43] reinforce the idea that mHealth can modulate internal health markers (lipids, glucose) even when BMI changes are modest [43,77]. Notably, Audi et al. [55] showed that digital dietary self-monitoring led to significantly more pronounced improvements in insulin resistance (HOMA-IR) and HDL-cholesterol compared to traditional paper-based methods, while also reducing scores on the Binge Eating Scale (BES). These findings contribute to the emerging perspective of a “Beyond BMI” approach to evaluation of mHealth, where metabolic stability and the quality of behavioral shifts are recognized as primary indicators of clinical success.

### 4.3. Technological Innovations and Hybrid “Blended Care”

The inaccuracy of manual food logging remains a significant barrier in nutritional research. Emerging technologies, such as AI-based image recognition, represent a potential advancement in addressing this gap [78]. By automating dietary assessment and hiding sensitive data like calorie counts, these tools may help reduce the risk of triggering eating-related concerns in vulnerable adolescents [32,78]. The potential advantages of digital interfaces over traditional methods was further reinforced by Audi et al. [55], who demonstrated that mobile-based self-monitoring yielded significantly better metabolic stabilization (HOMA-IR and HDL-c) than paper records, likely due to reduced user friction and the mitigation of weight-related stigma during the tracking process. Furthermore, the study by Mameli et al. [79] explored the synergy between energy expenditure data from wristbands and energy intake from smartphone applications; however, despite the personalized feedback, this technological combination was not found to be superior to standard care for weight loss [79]. Beyond passive monitoring, Staiano et al. [80] demonstrated that home-based exergaming, when paired with telehealth coaching, significantly reduced BMI z-scores and improved systolic blood pressure and lipid profiles (LDL-C and total cholesterol). Their findings suggest that gamified physical activity may be associated with high adherence (94.4%) and serve as a potent tool for enhancing cardiometabolic health in children with obesity [80]. A central theme across successful trials is the superiority of hybrid models. The “Human-in-the-Loop” may provide additional accountability that automated systems lack. This “digi-physical” synergy reached its most robust validation in the 3-year trial by Hagman et al. [53], where integrating digital tools into standard clinical care doubled the obesity remission rate compared to traditional treatment. As Umano et al. demonstrated, the app acts as a “retention glue,” keeping families connected to the clinician between visits [38,48]. This role of digital tools as a ‘clinical extender’ is not a novel phenomenon but rather the maturation of a principle established in early eHealth research. Over a decade ago, early research identified that even low-frequency electronic prompts could serve as a ‘behavioral tether,’ maintaining participant focus and reducing attrition in pediatric weight management programs. This integrated approach mirrors the Connect for Health model, which early demonstrated the efficacy of linking primary care with community-based digital support to improve both BMI and family-centered outcomes [81]. Further analysis of this model by Bala et al. underscored the high feasibility of such interventions, reporting that 93% of parents engaged with interactive text messaging and 97% expressed satisfaction with the digital community resource tools, highlighting telehealth as a potent vehicle for sustained family engagement [82]. The utility of integrated models is further evidenced by Fleischman et al., who showed that combining primary care with specialist tele-visits (dietitians/psychologists) significantly reduced BMI z-scores and maintained high retention (80–90%) by bridging the gap to multidisciplinary expertise [83]. Luque et al. showed that Obemat 2.0 multicomponent intervention supports this synergy through the integration of motivational interviewing, eHealth monitoring, and group sessions, offering a scalable ‘window of opportunity’ for longitudinal support in primary care [84]. This principle has been significantly refined through subsequent feasibility and process evaluations; for instance, Browne et al. demonstrated that the ‘process outcomes’ of mobile apps—specifically their ability to provide structured support—are critical for the successful delivery of multicomponent interventions [85]. Similarly, Naets et al. highlighted that digital self-control training can effectively address adherence barriers during multidisciplinary treatment, reinforcing the app’s role as a clinical extender [86]. For the clinical community, this implies that mHealth should extend the reach of the healthcare provider through ‘digital nudges’ and integrated feedback loops that sustain motivation and prevent the ‘engagement gap’ often seen in traditional settings [39,87,88]. As noted by Zhu et al. [51], the inclusion of weekly phone coaching alongside a web-based program is the essential “human element” that prevents mHealth fade-out, ensuring that technological innovation translates into sustained clinical efficacy.

### 4.4. Bridging the Gap: Digital Equity and Geographic Accessibility

The COVID-19 pandemic acted as a stress test for pediatric digital care, revealing both its resilient infrastructure and its systemic limitations [25,89]. A critical finding in this context is the capacity of mHealth to overcome geographical barriers. The study by Davis et al. demonstrated that telehealth can be particularly effective in rural areas, where the scarcity of pediatric obesity specialists often leads to treatment desertification [49]. By bringing high-intensity interventions directly into the home, digital platforms mitigate the logistical and financial burdens (e.g., travel time and costs) that traditionally hinder rural families’ participation [49,90]. As Audi et al. [55] observed, even when a clinic is available, a greater distance to the facility remains a primary predictor of dropout, a barrier that mobile interfaces effectively circumvent by fostering adolescent autonomy and remote monitoring.

However, “digital equity” extends beyond rural–urban divides within high-income countries. A pivotal finding from Wen et al. [54] suggests that mHealth may exert a more pronounced effect on historically underserved populations; in their 5-year extension study, nurse-led telehealth and SMS support yielded significantly stronger BMI reduction in low-income families compared to the general cohort (mean difference −0.57 vs. −0.30). This “inverse care law” reversal is supported by Lee et al. [46], who demonstrated that asynchronous eHealth models are highly accessible for low-income dyads, effectively overcoming literacy and scheduling barriers that often plague traditional in-person care. This underscores that mHealth, when designed with inclusivity, may act as a “socioeconomic equalizer” rather than a divider [60,90].

Despite these regional successes, a profound global imbalance remains in mHealth research. As highlighted by Reddy et al. [91], while European landscapes—represented in this review by the maturing evidence base in Sweden [28,36,53] and France [52]—show significant progress, data from low- and middle-income regions remain critically scarce despite high mobile phone penetration [91,92]. This suggests that while mHealth has the inherent potential to bridge health disparities, its implementation must be intentionally inclusive and culturally adapted—as seen in the multi-language success of the MINISTOP 2.0 trial [47] to move from localized efficacy to global public health impact [54].

### 4.5. Sustainability and Neurocognitive Frontiers

The transition from short-term ‘pilot’ enthusiasm to long-term ‘maintenance’ remains an important gap in the current pediatric mHealth literature [61,85]. While the recently published landmark 3-year data from Hagman et al. provides a rare longitudinal benchmark for success in specialized clinical settings, the majority of contemporary trials—including those analyzed in this review—continue to grapple with the ‘mHealth Fade-out’ effect [45,53]. This phenomenon, characterized by a precipitous decline in digital engagement after the initial 6-month ‘novelty’ phase, is a persistent barrier across diverse technological platforms [22,56,57]. As highlighted by Talens et al., although mobile interventions are highly effective at promoting acute dietary shifts, their long-term impact on BMI sustainability is frequently undermined by this attrition, a challenge previously echoed in earlier process evaluations of multicomponent digital tools [85,87].

To move beyond the limitations of passive monitoring, future research may benefit from incorporating neurocognitive and self-regulatory mechanisms. Naets et al. emphasized that digital tools focusing on executive functions and inhibitory control training may help address underlying psychological barriers to adherence more effectively than traditional calorie tracking [86]. This is further reinforced by Audi et al. [55], whose findings suggest that mobile interfaces may mitigate the “stigma-driven” barriers to self-monitoring, resulting in significant improvements in the Binge Eating Scale (BES) and metabolic health (HOMA-IR) even when weight loss is modest. Furthermore, emerging evidence suggests that smartphone-delivered attention retraining may influence neural reward processing for high-calorie foods [93]. Such neurobiological pathways to sustainability offer a more robust intervention model than simple data collection, potentially aligning with the sustained behavioral shifts observed in long-term school-based interventions like the ATLAS trial [69].

The findings of our meta-analysis suggest that mHealth interventions are associated with a modest but statistically significant reduction in BMI z-score in pediatric populations. However, the magnitude of the effect is relatively small and appears to be context-dependent. The fact that the prediction interval crosses the null value indicates that future studies may yield heterogeneous results, including minimal or no effect in certain settings.

This variability likely reflects differences in intervention design, parental adherence, and digital implementation. Taken together, these results support the potential role of mHealth in pediatric weight management while highlighting the critical importance of user engagement. Future research should prioritize high-quality RCTs with standardized outcome reporting and long-term follow-up to better assess the sustainability and generalizability of these digital solutions.

### 4.6. Synthesis of Lessons Learned and Future Directions

This meta-analysis suggests that mHealth interventions have a statistically significant, albeit modest, effect on reducing BMI z-score in children and adolescents. These findings support the growing body of evidence suggesting that digital health tools can play a supportive role in pediatric weight management. The moderate heterogeneity observed across studies likely reflects underlying clinical and methodological variability, including differences in participant age, intervention design, and duration. Variations in engagement and adherence may contribute to the observed inconsistency of effects, particularly in digital interventions where sustained user interaction plays a key role. Consistent with our findings, the meta-analysis by Jihyun Park et al. highlighted that ICT interventions may not show significant overall effects; however, meaningful BMI reductions were observed in specific subgroups, particularly in web-based interventions and among children with obesity, suggesting that intervention design and target population are critical determinants of effectiveness [94]. Similarly, the meta-analysis by Li-Ting Qiu et al. demonstrated that eHealth-delivered lifestyle interventions significantly improved multiple anthropometric outcomes, with greater effects observed in interventions incorporating parental or school involvement, longer duration, and mobile-based delivery, further supporting the importance of intervention structure and intensity [60].

A key limitation of the current evidence base is the limited use of direct body composition measures. Although parameters such as fat mass, body fat percentage, and lean mass are more closely associated with metabolic risk than BMI alone, only a small proportion of included trials reported such outcomes. The majority of studies relied on BMI-based metrics or surrogate anthropometric indicators (e.g., waist circumference or waist-to-height ratio), which may not accurately reflect changes in adiposity or body composition, as observed in several trials including those by Karssen et al. and Lee et al. [45,46].

This gap was particularly evident in early childhood interventions, where studies predominantly focused on behavioral outcomes and BMI-derived indicators, without assessing body composition, as reported by Lee et al. and Wen et al. [46,54]. Even large-scale and long-term trials, such as Hagman et al. [53], relied exclusively on BMI outcomes. Furthermore, in some studies, body composition was mentioned in the methodology but not reported in the results, as observed in Salahshoornezhad et al. [43], suggesting inconsistencies in outcome reporting.

Among the few studies that assessed body composition, findings were heterogeneous. While Tsai et al. [29] reported a significant reduction in body fat percentage, other trials, such as Mateo-Orcajada et al. [50], did not observe significant improvements despite intervention exposure. Similarly, Stasinaki et al. [40] reported improvements in body composition parameters without corresponding sustained changes in BMI. These findings suggest that while mHealth interventions may influence behavioral and metabolic pathways, their impact on adiposity remains insufficiently characterized. However, the observed heterogeneity indicates that not all interventions are equally effective. Differences in intervention design, including the level of personalization, behavioral support, and user engagement, may explain the variability in outcomes. Interventions incorporating interactive components or ongoing feedback may be more effective than passive or standalone applications. Further evidence is provided by the meta-analysis by Cheng-Tai Wu et al. showed that teleinterventions can significantly improve BMI and waist circumference in pediatric populations, with stronger effects observed in the short term and when interventions include family engagement and structured professional interaction [57].

The prediction interval further highlights that some interventions may yield little or no benefit, emphasizing the importance of optimizing intervention strategies.

Importantly, publication bias analyses suggested the potential presence of missing studies, and adjusted estimates indicated a reduction in effect size, with loss of statistical significance, while fail-safe N results further suggested the limited robustness of the findings. These observations are consistent with the systematic review and meta-analysis by Cheryl A. Margetin et al., which reported only a small effect of telehealth interventions on BMI z-score and low strength of evidence overall [95]. Taken together, these findings indicate that although mHealth interventions are promising, their true effect may be overestimated and should be interpreted with caution. Future research should therefore focus on high-quality randomized controlled trials with standardized outcome reporting and longer follow-up to better assess the sustainability and generalizability of these interventions.

Several limitations should be considered when interpreting these findings. First, the relatively small number of studies included in the meta-analysis limits statistical power and reduces confidence in the pooled estimates. Second, the presence of moderate heterogeneity (*I*^2^ = 60%) indicates substantial between-study variability, likely driven by differences in population characteristics, intervention design, and follow-up duration. Third, risk of bias was a concern in several studies, particularly due to missing outcome data and reliance on self-reported measures, which may have led to overestimation of intervention effects. Fourth, high dropout rates observed in multiple trials further reduce the reliability and generalizability of the findings, especially in adolescent populations where engagement was inconsistent. Finally, publication bias cannot be excluded, and adjusted analyses suggested that the observed effect size may be overestimated. Taken together, these limitations indicate that the results should be interpreted with caution. The qualitative synthesis of the 23 included RCTs shown in Table 5 suggests a shift in pediatric obesity management: the transition from ‘digital isolation’ to ‘integrated connectivity’. By mapping the 18 included RCTs, we observed that interventions over-relying on passive tracking or isolated gamification, such as those by Mateo-Orcajada et al. and Tugault-Lafleur, were associated with higher attrition and the “boredom factor” [32,50]. In contrast, the “active ingredients” identified in the most successful trials—specifically Hagman et al. and Thorén et al.—tend to favor a “blended care” approach where technology serves as a clinical extender. This aligns with broader process evaluations suggesting that without intrinsic motivation or social support, digital tools may fail to transition from novelty to habit formation [75,85,96]. In contrast, the current evidence base—exemplified by Thorén et al. and Delli Bovi—tends to favor a blended care approach where technology serves as a clinical extender, reinforcing the therapeutic alliance between face-to-face consultations [48,87].

This synthesis suggests that the “active ingredients” of successful interventions may not be purely technological but also relational. As demonstrated by Umano et al. [48], mHealth may act as a “retention glue” that stabilizes families within the healthcare system. This role may be important, as previous longitudinal studies have shown that clinic attendance is one of the strongest predictors of long-term weight stabilization [87,97]. Additionally, the blended program evaluated by Perdew et al. demonstrated that while BMI z-scores remained unchanged over 10 weeks, the intervention significantly strengthened parental self-regulation and support for physical activity, providing the behavioral framework necessary for long-term success [98]. Furthermore, the disconnect observed in trials like Memarian et al. [44]—where behavioral shifts occurred without immediate BMI changes—supports the potential role of mHealth as a catalyst for parental self-efficacy and pediatric habit formation. These “pre-anthropometric” shifts may represent important precursors to long-term success, as documented in foundational behavioral interventions [69,86,99].

Moving forward, the gaps identified in Table 5—specifically the “mHealth fade-out” and the maturity requirements for adolescent autonomy—suggest that the next generation of interventions may need to evolve [52]. Beyond simple calorie counting, future designs could incorporate neurocognitive retraining, AI-driven personalization, and IoT-integrated monitoring to provide objective, real-time feedback [93,100,101,102]. By bridging the gap between clinical expertise and the daily home environment [57,58], mHealth may provide a framework for a resilient digital health ecosystem that may remain effective during global disruptions [22,29,56,61,63]. Ultimately, as we move toward 2030, these technologies may be considered for integration into future pediatric guidelines to ensure that digital empathy and clinical precision work in tandem to address the multifaceted nature of childhood obesity [18,53].

## 5. Conclusions

This systematic review suggests that mHealth interventions are most effective when integrated into a “blended care” framework, where digital tools complement rather than replace professional clinical oversight. Standalone applications often struggle with long-term engagement; however, when combined with human accountability, they act as a vital “retention bridge” between clinical visits, stabilizing weight trajectories even during global disruptions. While mHealth interventions are associated with a modest but statistically significant reduction in BMI z-score in pediatric populations, variability in effectiveness and potential publication bias highlight the need for cautious interpretation. Consequently, further well-designed studies are required to determine the most effective components of these interventions and their long-term impact.

Although significant BMI reduction was achieved in over half of the analyzed trials, the most consistent clinical value of mHealth lies in the successful “re-patterning” of lifestyle behaviors. Digital tools proved superior in modifying dietary habits and reducing sedentary time—changes that often precede anthropometric shifts—provided that the intervention design actively mitigates the “fade-out” effect through gamification or interactive feedback. Ultimately, the future of pediatric obesity management depends on moving away from a “one-size-fits-all” approach toward developmentally tailored ecosystems. To ensure sustainability and scalability, next-generation protocols must differentiate between parental empowerment in early childhood and adolescent autonomy, utilizing emerging technologies like AI to reduce the burden of self-monitoring and secure long-term adherence. These findings support the integration of mHealth strategies as complementary tools within multidisciplinary approaches to pediatric obesity, necessitating a clinical vision that looks beyond BMI.

## Figures and Tables

**Table 1:** Literature Search Strategy Overview.

Component	Details
Keywords	“Childhood Obesity”, “Pediatric obesity”, “Paediatric obesity”, “eHealth”, “mHealth”, “Smartphone”, “app”, “Mobile phone”, “Randomized controlled trial”
Databases Searched	PubMed, Web of Science, Scopus, Lens.org, Dimensions.ai
Timeframe	1 January 2020 to 29 January 2026; additional Scopus update on 28 March 2026
Language	English
Study Types Included	Randomized Controlled Trials
Additional Sources	References cited in included articles and selected reviews
Inclusion Criteria	- Pediatric population (0–18 years); mHealth/eHealth intervention; anthropometric or metabolic outcomes; follow-up assessment
Exclusion Criteria	- Reviews, meta-analyses, observational studies, case reports, editorials, conference abstracts without full text, non-English studies, adult populations, animal or laboratory-based studies

Legend: eHealth = electronic Health; mHealth = mobile Health.

**Table 2:** Risk of Bias Assessment (RoB 2) of the Included Studies.

Study ID	D1	D2	D3	D4	D5	Overall	Justification for Overall Rating
Johansson et al. (2020) [36]	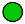	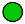	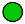	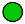	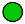	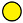	High methodological quality.
Likhitweerawong et al. (2020) [37]	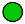	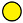	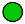	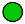	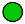	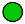	High methodological quality for a behavioral intervention trial.
Delli Bovi et al. (2021) [38]	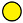	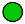	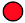	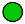	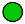	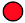	Quasi-experimental allocation; high dropout in control (62%).
Chai et al. (2021) [39]	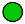	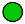	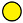	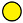	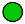	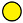	22% attrition; reliance on parent-reported dietary data (FFQ).
Stasinaki et al. (2021) [40]	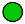	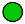	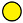	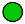	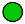	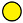	Significant dropout between recruitment and intervention start.
Vidmar et al. (2022) [41]	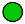	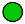	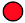	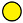	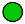	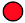	High attrition (42%); BMI measurements disrupted by pandemic.
Liu et al. (2022) [42]	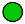	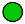	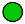	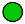	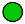	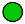	Rigorous cluster-RCT with 97.8% retention and objective metrics.
Salahshoornezhad et al. (2022) [43]	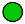	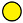	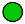	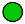	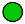	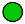	Balanced randomization and 0% attrition; unblinded.
Memarian et al. (2022) [44]	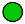	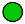	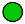	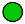	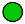	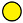	Loss of blinding at 3-month follow-up potentially biased long-term data.
Karssen et al. (2022) [45]	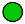	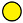	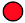	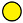	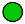	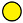	High missingness for zBMI (64.9% at 12 mo); parent-reported data.
Lee (2023) et al. [46]	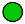	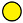	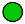	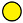	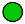	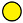	The “Some Concerns” rating is primarily due to the self-reported nature of the behavioral metrics and the lack of participant blinding.
Tugault-Lafleur et al. (2023) [32]	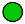	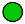	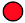	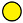	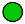	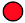	37% attrition; significant differential loss in intervention group.
Alexandrou et al. (2023) [47]	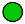	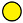	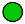	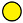	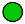	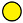	Primary behaviors self-reported; objective BMI measured by nurses.
Tsai et al. (2024) [29]	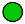	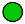	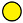	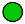	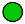	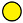	High attrition (47%) due to COVID-19, balanced by active placebo design.
Thorén et al. (2024) [28]	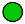	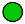	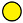	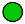	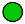	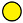	12% missing data at 6 mo; waitlist effect may influence control.
Umano et al. (2024) [48]	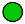	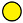	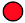	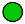	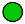	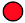	Critical dropout in control group (72%) vs. intervention (40%).
Davis et al. (2024) [49]	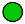	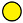	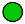	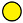	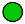	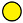	Switch to home-based/Zoom measures due to COVID-19.
Mateo-Orcajada et al. (2024) [50]	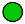	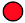	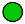	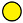	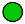	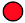	Major adherence failure (>50% stopped app use after week 6).
Zhu et al. (2025) [51]	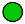	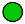	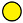	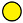	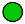	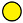	~31% attrition; reliance on parent-reported measurement kits.
Foissac et al. (2025) [52]	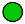	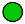	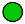	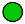	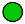	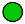	High retention (92%) and objective clinical measurements.
Hagman et al. (2025) [53]	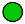	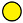	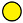	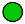	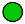	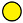	Due to the pragmatic trial nature and significant long-term attrition.
Wen et al. (2025) [54]	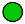	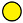	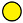	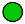	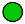	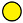	High attrition rates and lack of participant blinding are notable concerns.
Audi et al. (2026) [55]	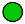	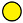	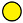	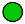	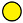	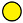	Primarily due to high attrition and retrospective trial registration.

Legend: D1: Bias arising from the randomization process; D2: Bias due to deviations from intended interventions; D3: Bias due to missing outcome data; D4: Bias in measurement of the outcome; D5: Bias in selection of the reported result. Judgment symbols: 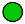 Low risk of bias; 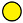 Some concerns; 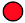 High risk of bias. Abbreviations: *N*: Sample size (number of participants); RCT: Randomized Controlled Trial; BMI: Body Mass Index; zBMI: BMI z-score (Standard Deviation Score); FFQ: Food Frequency Questionnaire; mo: months; vs.: versus; ~: approximately.

**Table 3:** Summary of Study Characteristics and mHealth Intervention Designs.

Study ID	Country	Population (*N*; Intervention/Control; Age; Weight Status)	Intervention (mHealth Component)	Comparison	Duration	Study Design
Johansson et al. (2020) [36]	Sweden	*N* = 28 (14/14); 5–12 y; OB	Provement: digital scales + parent app	Usual care (clinic visits)	6 mo	Pilot RCT
Likhitweerawong et al. (2020) [37]	Thailand	*N* = 77 (38/39); 10–15 y; OB (BMI ≥95th)	OBEST app: self-monitoring, goals, education, clinician messaging	Standard care	2 mo	RCT
Delli Bovi et al. (2021) [38]	Italy	*N* = 103 (51/52); 6–14 y; OB (BMI >95th)	PediaFit: WhatsApp + hybrid sessions	Usual care	6 mo	RCT
Chai et al. (2021) [39]	Australia	*N* = 46 (22/24); 4–11 y; OW/OB risk	Telehealth: web + Facebook + SMS	Waitlist	12 wk	Pilot RCT
Stasinaki et al. (2021) [40]	Switzerland	*N* = 41 (21/20); 10–18 y; OB	PathMate2: chatbot + biofeedback	Face-to-face intervention	12 mo	RCT
Vidmar et al. (2022) [41]	USA	*N* = 117 (39/38/40); 14–18 y; OB	AppAlone vs. AppCoach model	Multidisciplinary care	24 wk	3-arm RCT
Liu et al. (2022) [42]	China	*N* = 1392 (691/701); 8–10 y	School policy + monitoring app	Usual practice	9 mo	Cluster RCT
Salahshoornezhad et al. (2022) [43]	Iran	*N* = 62 (31/31); 9–12 y (girls); OW/OB	Gamified nutrition + CBT	Traditional lectures	10 wk	RCT
Memarian et al. (2022) [44]	Iran	*N* = 46 (23/23); 7–11 y; OW/OB	Gamified “Go/No-Go” app	Active control	3 mo	RCT
Karssen et al. (2022) [45]	The Netherlands	*N* = 357 (181/176); 5–15 mo; infants	Samen Happie! parenting app	Waitlist	12 mo	RCT
Lee et al. (2023) [46]	USA	*N* = 73 (37/36); 1–3 y; low-income; OB risk	Videos + SMS + education	Printed newsletters	8 wk	Pilot RCT
Tugault-Lafleur et al. (2023) [32]	Canada	*N* = 214 (107/107); 10–17 y; OW/OB	Aim2Be: gamified app + coach	Waitlist (brochure)	3–6 mo	RCT
Alexandrou et al. (2023) [47]	Sweden	*N* = 552 (276/276); 2.5–3 y	MINISTOP 2.0 parent app	Usual care + brochure	6 mo	RCT
Tsai et al. (2024) [29]	Taiwan	*N* = 164 (82/82); 6–9 y; OW/OB	Education + monthly SMS	Active placebo	12 mo	RCT
Thorén et al. (2024) [28]	Sweden	*N* = 65 (34/31); 5–12 y; OB	Web-COP + group sessions	Waitlist	6 mo	RCT
Umano et al. (2024) [48]	Italy	*N* = 75 (38/37); 6–12 y; OB	Nutrilio app + feedback	Usual care	12 mo	RCT
Davis et al. (2024) [49]	USA	*N* = 148 (74/74); 6–10 y; OW/OB	Telehealth (video sessions)	Newsletters	20 mo	Cluster RCT
Mateo-Orcajada et al. (2024) [50]	Spain	*N* = 50 (25/25); 12–16 y; OW/OB	Strava step-tracking	Standard PE	10 wk	RCT
Zhu et al. (2025) [51]	Australia	*N* = 102 (51/51); 7–13 y; OW/OB	Web program + phone coaching	Waitlist	10 wk	RCT
Foissac et al. (2025) [52]	France	*N* = 78 (39/39); 11–17 y; severe OB	App-based monitoring	Standard care	15 mo	RCT
Hagman et al. (2025) [53]	Sweden	*N* = 428 (107/321); 4–17.9 y; OB	Digital tools + clinic visits	Standard care	3 y	RCT
Wen et al. (2025) [54]	Australia	*N* = 662 (331/331); 2–5 y	Phone + SMS + materials	Printed materials	2 y + 1 y follow-up	RCT
Audi et al. (2026) [55]	Brazil	*N* = 60 (30/30); 13–17 y; OB	FatSecret app + diet	Paper food record	6 mo	Pilot RCT

Legend: OB: Obesity; OW: Overweight; CBT: Cognitive Behavioral Therapy; PE: Physical Education; mo: months; wk: weeks; y: years; RCT: Randomized Controlled Trial.

**Table 4:** Clinical Outcomes, Retention & Engagement.

Study ID	Key Clinical Outcomes (Anthropometric/Behavioral)	Study Focus	Retention Rate	mHealth Engagement
Johansson et al. (2020) [36]	BMI SDS reduced in intervention vs. control (*p* = 0.002).	Objective monitoring	89%	80% used digital scales regularly
Likhitweerawong et al. (2020) [37]	BMI reduced in intervention; weight increased in control (*p* = 0.005); improved psychosocial scores (*p* = 0.049).	Weight and psychosocial outcomes	92% vs. 90%	31% ≥ 50% compliance
Delli Bovi et al. (2021) [38]	zBMI reduced (*p* = 0.04); increased fruit/vegetable intake (*p* = 0.02).	Hybrid care	90% vs. 38%	100% messaging feedback
Chai et al. (2021) [39]	Reduced energy-dense food intake (*p* = 0.038); no BMI difference.	Telehealth + SMS	78%	96% session attendance
Stasinaki et al. (2021) [40]	Reduced body fat (*p* < 0.05); no BMI-SDS difference.	Chatbot-based intervention	94% starters	71.5% daily use
Vidmar et al. (2022) [41]	Reduced food addiction symptoms (*p* = 0.045); no BMI change.	Behavioral model	58%	~11 h app use
Liu et al. (2022) [42]	BMI reduced (*p* < 0.001); obesity prevalence reduced by 27%.	School-based intervention	97.8%	Higher use linked to greater effect
Salahshoornezhad et al. (2022) [43]	BMI reduced vs. control (*p* = 0.01).	Gamified CBT	100%	100% session compliance
Memarian et al. (2022) [44]	Reduced sweet intake (*p* < 0.01); no BMI change.	Cognitive training	>93%	>80% adherence
Karssen et al. (2022) [45]	zBMI reduced at 6 months (*p* < 0.001); no effect at 12 months.	Infant prevention	88%	Decline in app use
Lee et al. (2023) [46]	Increased fruit/vegetable intake (*p* < 0.01); reduced screen time (*p* = 0.026); no zBMI difference.	Early prevention	93%	High video engagement
Tugault-Lafleur et al. (2023) [32]	No zBMI difference (*p* = 0.51); reduced screen time in control (*p* = 0.003).	Gamified app	63%	<50% app use by week 2
De-Jongh González (2022) [33] (Secondary analysis)	Secondary analysis showed significant decrease only in ‘Fully Engaged’ parents.	Gamified app	63%	<50% app use by week 2
Alexandrou et al. (2023) [47]	Reduced sweet drink intake (*p* < 0.001); no BMI difference.	Real-world intervention	93%	54% weekly use
Tsai et al. (2024) [29]	zBMI lower at 12 months (*p* = 0.03); reduced body fat.	Family-based mHealth	53%	SMS-based engagement
Thorén et al. (2024) [28]	zBMI reduced vs. control (*p* < 0.001).	Hybrid intervention	87%	74% parental engagement
Umano et al. (2024) [48]	No BMI difference; reduced attrition (*p* = 0.01).	Parent-focused intervention	60%	60% compliance
Davis et al. (2024) [49]	zBMI improved at 20 months (*p* = 0.048).	Rural telehealth	>87%	15.8 h completed
Mateo-Orcajada et al. (2024) [50]	Improved fitness; no BMI change.	Passive tracking	83%	>50% app dropout
Zhu et al. (2025) [51]	zBMI reduced (*p* = 0.018).	Web + coaching	69%	90% module completion
Foissac et al. (2025) [52]	No zBMI difference (*p* = 0.86); similar success rates (~30%).	Maintenance app	92%	High engagement
Hagman et al. (2025) [53]	zBMI reduced (*p* = 0.02); higher remission (*p* = 0.0046).	Long-term hybrid care	58% vs. 45%	Frequent home monitoring
Wen et al. (2025) [54]	BMI reduced (*p* = 0.039); stronger in low-income groups (*p* = 0.018).	Early prevention	81% → 61%	58–64% session participation
Audi et al. (2026) [55]	zBMI reduced in both groups; improved HOMA-IR (*p* = 0.008) and HDL-c (*p* = 0.023).	Dietary self-monitoring	60% vs. 53.3%	26.7–53.3% adherence

Legend: BMI: Body Mass Index; zBMI: BMI z-score; SDS: Standard Deviation Score; CBT: Cognitive Behavioral Therapy.

**Table 5:** Assessment of Body Composition Outcomes and Measurement Methods in Included RCT.

Study	Body Composition Outcome	Measurement	Result	Notes
Johansson et al. (2020) [36]	Anthropometric measures only	BMI-SDS	No body composition assessed	Only BMI SDS used
Likhitweerawong et al. (2020) [37]	Anthropometric measures only	Waist circumference	No significant change	No direct body composition
Delli Bovi et al. (2021) [38]	Anthropometric measures only	BMI/zBMI	No body composition assessed	Behavioral + BMI outcomes
Chai et al. (2021) [39]	Anthropometric measures only	BMI, zBMI, waist circumference	No significant changes	Behavioral improvements
Stasinaki et al. (2021) [40]	Reported	BIA (fat mass, muscle mass)	Significant improvement	No sustained BMI-SDS reduction
Vidmar et al. (2022) [41]	Anthropometric measures only	BMI	No association	Behavioral focus
Liu et al. (2022) [42]	Reported	DXA + MRI	Fat ↓, lean mass ↑	Not mHealth; demonstrates the importance of body composition
Salahshoornezhad et al. (2022) [43]	Anthropometric measures only	BMI, WC, HC, WHR	Significant improvement	Body composition not reported
Memarian et al. (2022) [44]	Anthropometric measures only	BMI	No BMI change	Behavioral only
Karssen et al. (2022) [45]	Anthropometric measures only	zBMI (weight/length)	Not reported	Infant population; no body composition
Lee et al. (2023) [46]	Anthropometric measures only	BMI-for-age z-score	Not reported	Behavioral outcomes focus
Tugault-Lafleur et al. (2023) [32]	Anthropometric measures only	zBMI	No significant effect	No body composition measures
Alexandrou et al. (2023) [47]	Anthropometric measures only	BMI	No significant effect	Body composition only in previous trial (MINISTOP 1.0)
Tsai et al. (2024) [29]	Reported	Skinfold thickness → % body fat	Significant reduction in fat %	Also improved BMI and zBMI
Thorén et al. (2024) [28]	Anthropometric measures only	BMI, BMI-SDS, WC	Not reported	No body composition measures
Umano et al. (2024) [48]	Anthropometric measures only	Waist-to-height ratio	No significant differences	Proxy adiposity only
Davis (2024) [49]	Anthropometric measures only	BMI z-score	Not reported	BMI only
Mateo-Orcajada et al. (2024) [50]	Reported	Kinanthropometry (skinfolds)	No significant improvement	Short duration; small sample
Zhu et al. (2025) [51]	Anthropometric measures only	Waist circumference	No significant difference	Self-reported measurement
Foissac et al. (2025) [52]	Anthropometric measures only	BMI	Reduction observed	No body composition measures
Hagman et al. (2025) [53]	Anthropometric measures only	BMI, zBMI	Significant reduction	Long-term trial
Wen et al. (2025) [54]	Anthropometric measures only	BMI, zBMI	Significant reduction	No body composition measures
Audi et al. (2026) [55]	Reported	BIA (fat mass, lean mass)	Improved in both groups	No between-group difference

Legend: BMI: Body Mass Index; zBMI: BMI z-score; WC: waist circumference; HC: hip circumference; WHR: waist-to-hip ratio; BIA: bioelectrical impedance analysis; DXA: dual-energy X-ray absorptiometry; MRI: magnetic resonance imaging, RCT: Randomized Controlled Trial, ↓ decrease, ↑ increase.

**Table 6:** Qualitative Synthesis of Research Gaps, Critical Observations, and Future Directions for Pediatric mHealth Interventions.

Study ID	Main Finding	Research Gap	Observations
Johansson et al. (2020) [36]	Reduction in BMI SDS reported.	Small sample (*N* = 28); no long-term follow-up.	Objective measurements; clinician feedback.
Likhitweerawong et al. (2020) [37]	Reduction in BMI and improved psychosocial outcomes over 2 months.	Short duration; limited long-term adherence data.	Variable compliance; app-based self-monitoring.
Delli Bovi et al. (2021) [38]	Reduction in BMI z-score and screen time.	Quasi-experimental design; non-specialist staff.	Hybrid intervention; higher retention in intervention group.
Chai et al. (2021) [39]	No BMI change; improved dietary behaviors.	Small sample; short duration.	Telehealth and SMS components used.
Stasinaki et al. (2021) [40]	Improved body composition and fitness; no BMI-SDS change.	No association between stress reduction and BMI.	Chatbot-based intervention; high adherence.
Vidmar et al. (2022) [41]	Improved psychological outcomes; no BMI change.	No dietary intervention component.	Study conducted during COVID-19.
Liu et al. (2022) [42]	Reduction in BMI and obesity prevalence.	No significant increase in MVPA.	School-based and parent-supported intervention.
Salahshoornezhad et al. (2022) [43]	Improved BMI and metabolic parameters.	Short duration; small sample; female-only cohort.	Multidisciplinary intervention.
Memarian et al. (2022) [44]	Improved dietary behavior; no BMI change.	Limited dietary scope.	Gamified intervention.
Karssen et al. (2022) [45]	Short-term zBMI reduction not maintained at follow-up.	High missing data; limited long-term engagement.	Decline in app use over time.
Lee et al. (2023) [46]	Improved diet and reduced sedentary behavior; no BMI change.	Small sample; short duration.	Video-based intervention.
Tugault-Lafleur et al. (2023) [32]	No significant BMI or dietary differences.	Limited engagement effectiveness.	High attrition; low sustained app use.
Alexandrou et al. (2023) [47]	Improved diet and screen time; no BMI change.	Self-reported data; short duration.	Multilingual app; high retention.
Tsai et al. (2024) [29]	No additional BMI reduction vs. standard care.	High dropout in control group.	App use associated with lower attrition.
Thorén et al. (2024) [28]	Prevention of BMI increase compared to control.	Lower intervention dose than recommended.	Conducted in rural population.
Umano et al. (2024) [48]	No change in BMI or body composition.	Lack of interactive features.	High dropout; low engagement.
Davis et al. (2024) [49]	Reduction in BMI-SDS in intervention group.	Inability to isolate intervention components.	Parental involvement included.
Mateo-Orcajada et al. (2024) [50]	Stabilization of BMI z-score vs. increase in control.	No improvement in sleep outcomes.	Use of active control.
Zhu et al. (2025) [51]	Reduction in BMI z-score and improved quality of life.	Parent-reported anthropometric data; short follow-up.	Weekly coaching support.
Foissac et al. (2025) [52]	Non-inferiority to standard care for weight maintenance.	Reduced effect in younger adolescents.	App-based monitoring without parental involvement.
Hagman et al. (2025) [53]	Greater reduction in zBMI and higher remission rates vs. control.	Limited long-term data in diverse settings.	Combined digital and in-person care.
Wen et al. (2025) [54]	Reduction in BMI, stronger in low-income groups.	Limited cost-effectiveness data.	Phone and SMS-based intervention.
Audi et al. (2026) [55]	Similar zBMI reduction; improved metabolic parameters in app group.	Low-to-moderate adherence.	Digital dietary self-monitoring.

Legend: BMI/zBMI: Body Mass Index z-score; SDS: Standard Deviation Score; mHealth Fade-out: the phenomenon of rapid decline in digital engagement after the initial intervention phase; Hybrid Model: an intervention combining mobile technology with human professional support.

## Data Availability

No new data were created or analyzed in this study. Data sharing is not applicable to this article.

## Supplementary Materials

The following supporting information can be downloaded at: https://www.mdpi.com/article/10.3390/nu18101511/s1.
