# Supplementary material for: Beyond BMI: A Systematic Review and Meta-Analysis of mHealth Interventions for Pediatric Obesity Management
Source: Nutrients. 2026 May 9;18(10):1511. doi: 10.3390/nu18101511 (PMC13209689; doi:10.3390/nu18101511)
Supplement: Supplementary file 1 [file nutrients-18-01511-s001.zip › nutrients-4287216-supplementary/Supplementary material S3.docx]

A meta-analysis was performed using a random-effects model with the generic inverse variance method. Effect sizes were expressed as mean differences (MD) with corresponding 95% confidence intervals.

Standard errors were calculated from reported standard deviations or derived from confidence intervals when available. In cases where variance data were not reported, standard errors were conservatively approximated based on available information.

Between-study variance (τ²) was estimated using the restricted maximum likelihood (REML) method. Confidence intervals for the pooled effect were calculated using the Hartung-Knapp adjustment, providing a more conservative estimate appropriate for a limited number of studies.

Prediction intervals were calculated using a t-distribution approach to estimate the range of true effects in future studies.

| **Study** | **TE (Mean Difference)** | **seTE (Standard Error)** | **Weight (%)** | **Lower CI** | **Upper CI** |
| --- | --- | --- | --- | --- | --- |
| Johansson (2020) | -0.24 | 0.0765 | 22.8% | -0.39 | -0.09 |
| Zhu (2025) | -0.10 | 0.0459 | 28.5% | -0.19 | -0.01 |
| Liu (2022) | -0.46 | 0.1071 | 16.1% | -0.67 | -0.25 |
| Foissac (2025) | -0.02 | 0.1071 | 16.1% | -0.23 | 0.19 |
| Tsai (2024) | -0.31 | 0.1429 | 10.4% | -0.59 | -0.03 |
| Karssen (2022) | -0.15 | 0.1224 | 6.1% | -0.39 | 0.09 |


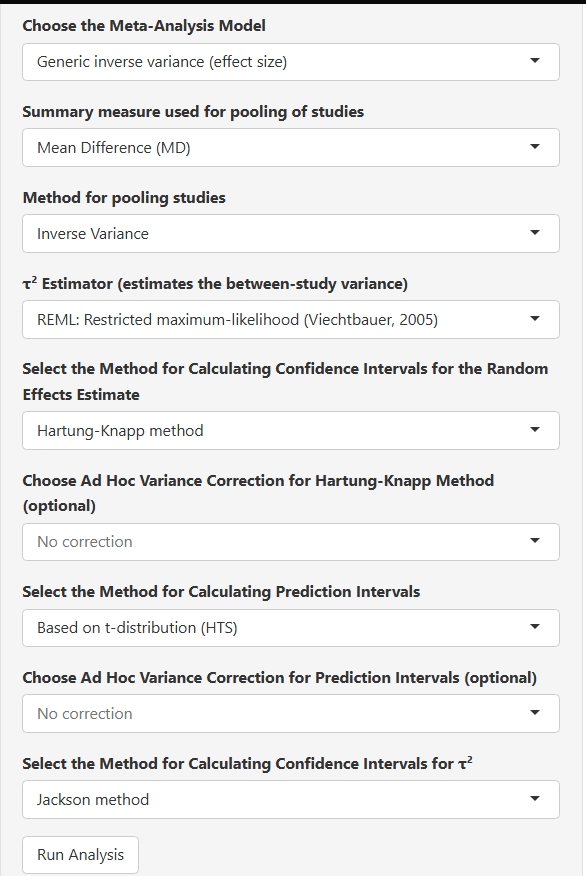


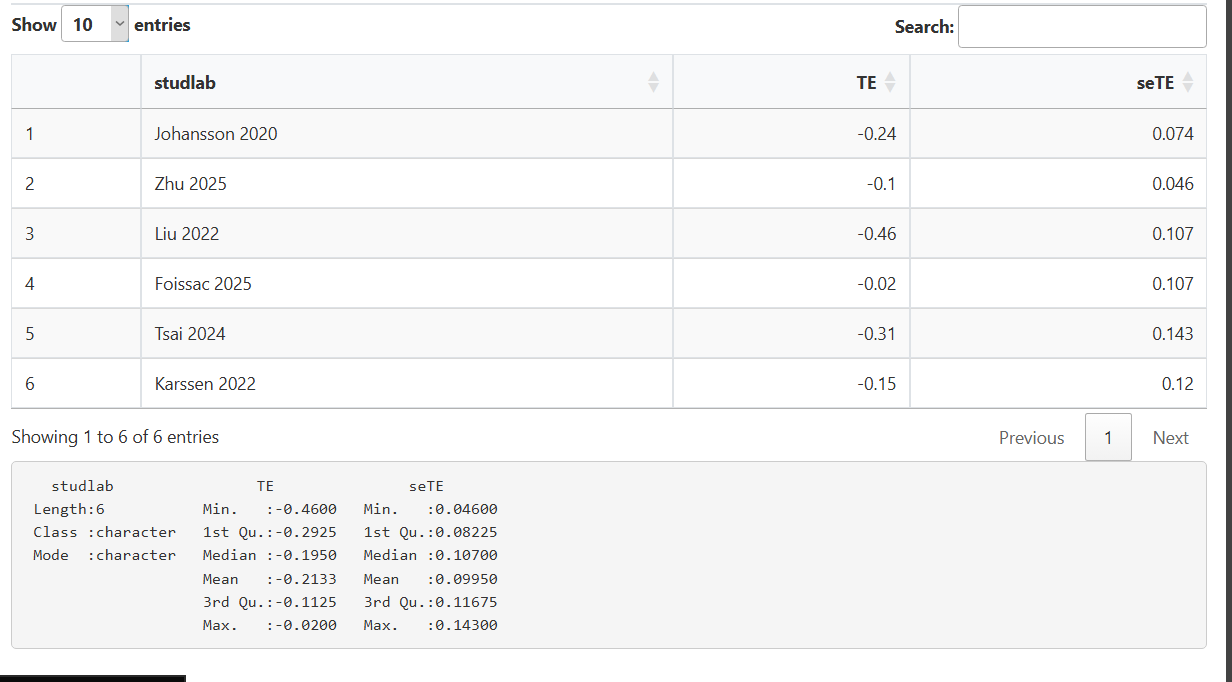


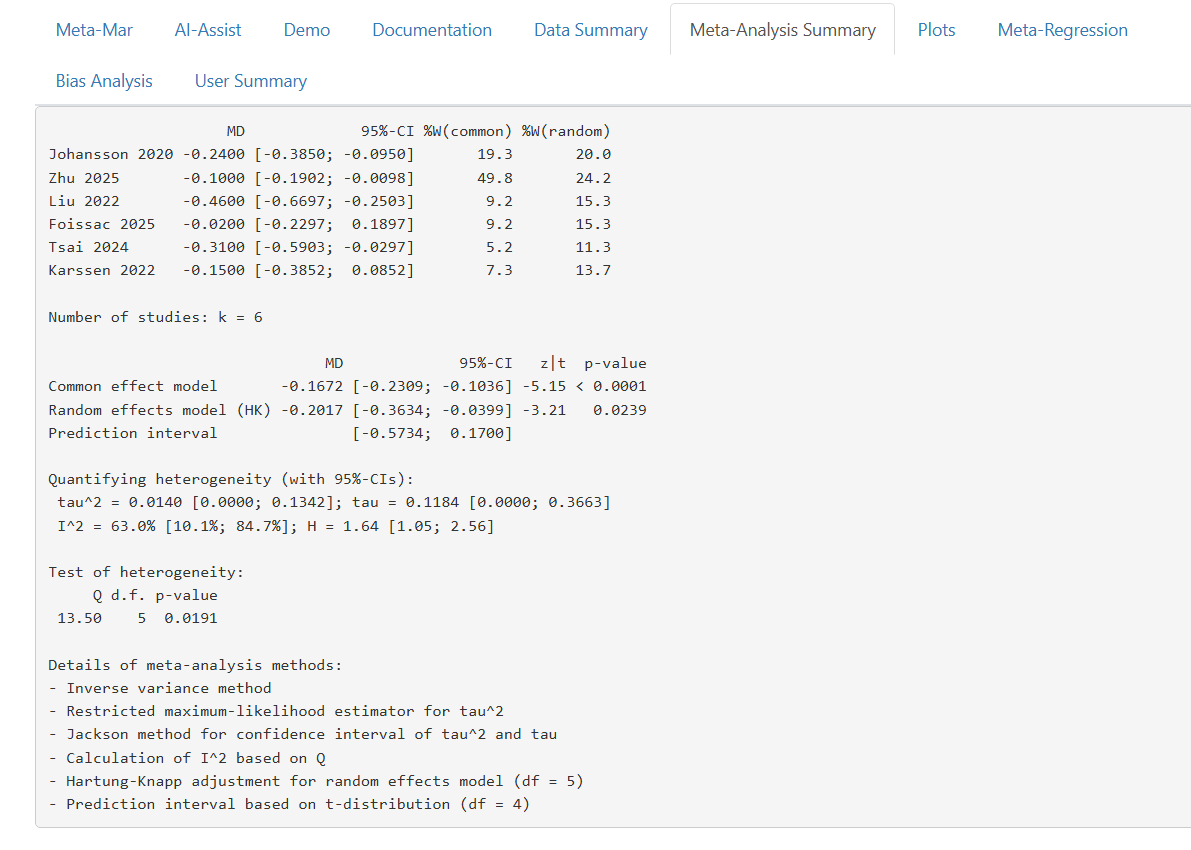


A random-effects model was used due to clinical and methodological heterogeneity across studies.

Heterogeneity was assessed using Cochran’s Q test and the I² statistic.

Moderate heterogeneity was observed (Q = 13.5, p = 0.019; I² ≈ 63%), indicating that variability between study results was not solely due to chance.


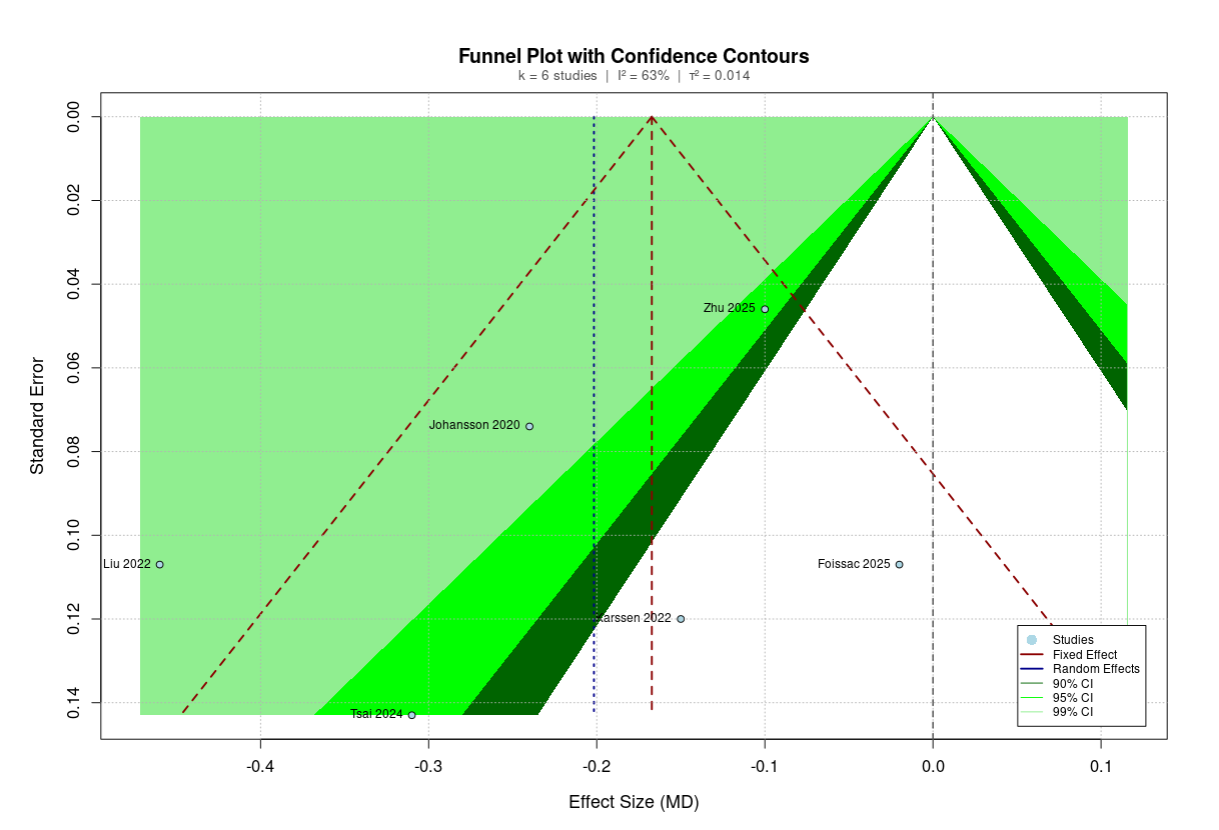


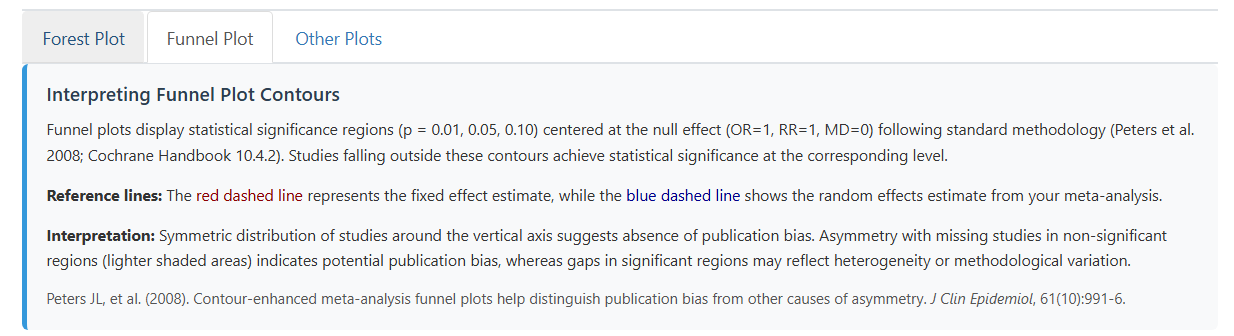


Publication bias analyses (trim-and-fill and fail-safe N) suggested potential bias; however, these results should be interpreted cautiously given the limited number of studies.

Funnel plot interpretation is limited due to the small number of included studies (n = 6), as recommended by Cochrane guidelines.

Publication bias analyses (trim-and-fill and fail-safe N) suggested potential bias; however, these findings should be interpreted cautiously due to limited statistical power.

# META-ANALYSIS SUMMARY REPORT -- Meta Mar

Generated on: 2026-03-26 02:26:34

## MODEL SETTINGS

--------------------------------------------------------

Model type: metagen: Generic inverse variance meta-analysis (effect size)

Summary measure: MD: Mean Difference

Tau estimator: REML: Restricted maximum-likelihood (Viechtbauer, 2005)

Random effects ci method: HK: Hartung-Knapp method - more conservative approach

Predict method: HTS: Based on t-distribution

Tau ci method: J: Jackson method for confidence intervals of τ²

Pooling method: Inverse Variance weighting

Adhoc hakn ci:

Adhoc hakn pi:

Number of studies: 6

## FIXED EFFECT MODEL RESULTS

--------------------------------------------------------

Effect size: -0.1672

95% CI: [-0.2309, -0.1036]

Z-value: -5.15

p-value: 2.604e-07

## RANDOM EFFECTS MODEL RESULTS

--------------------------------------------------------

Effect size: -0.2017

95% CI: [-0.3634, -0.0399]

Z-value: -3.2054

p-value: 0.02385

Prediction interval: [-0.5734, 0.17]

## HETEROGENEITY STATISTICS

--------------------------------------------------------

τ² (between-study variance): 0.014

I² (percentage of variability due to heterogeneity): 60% (moderate heterogeneity)

H (square root of the inflation factor): 1.64

Cochran's Q: 13.5, df = 5, p = 0.01911

Q test suggests significant heterogeneity (p ≤ 0.10)

## PUBLICATION BIAS ANALYSIS

--------------------------------------------------------

### Egger's Test for Funnel Plot Asymmetry

### Begg's Rank Correlation Test

### Thompson-Sharp Test

### Trim and Fill Analysis

Number of observed studies: 6

Estimated missing studies: 2

Total studies after trim-fill: 8

Original effect estimate (random effects): -0.2017 [-0.3634, -0.0399]

Adjusted effect estimate: -0.1285 [-0.3025, 0.0455]

Absolute change in effect: 0.0732

Percentage change in effect: 36.3%

Interpretation: The trim and fill method suggests the presence of 2 potentially missing studies.

The effect estimate changed substantially (>20%), indicating potential publication bias.

###Fail-Safe N Analysis

1. ROSENTHAL'S FAIL-SAFE N METHOD

-------------------------------

Fail-safe N Calculation Using the Rosenthal Approach

Observed Significance Level: 0.0484

Target Significance Level: 0.05

Fail-safe N: 1

Interpretation:

Rosenthal's fail-safe N estimates how many studies with null results would

need to be added to make the combined effect non-significant (p > 0.05).

The calculated fail-safe N is less than the threshold of 5k+10 = 40,

suggesting the results may be susceptible to publication bias.

2. ORWIN'S FAIL-SAFE N METHOD

---------------------------

Fail-safe N Calculation Using the Orwin Approach

Average Effect Size: -0.1923

Target Effect Size: -0.0962

Fail-safe N: 6

Interpretation:

Orwin's fail-safe N estimates how many studies with zero effect would

need to be added to reduce the effect size to a trivial level.

This indicates the robustness of your findings. A larger number suggests

that the effect is more robust against potential publication bias.

3. ROSENBERG'S FAIL-SAFE N METHOD

------------------------------

Fail-safe N Calculation Using the Rosenberg Approach

Average Effect Size: -0.1923

Observed Significance Level: 0.1095

Target Significance Level: 0.05

Fail-safe N: 0

Interpretation:

Rosenberg's fail-safe N estimates how many studies with null results would

be needed to reduce the combined effect to a non-significant level.

This provides an estimate of how robust your findings are against publication bias.

A larger number suggests greater robustness to potential publication bias.

• MD = −0.20

• 95% CI: −0.36 la −0.04

• p = 0.02
