# Supplementary material for: Beyond BMI: A Systematic Review and Meta-Analysis of mHealth Interventions for Pediatric Obesity Management
Source: Nutrients. 2026 May 9;18(10):1511. doi: 10.3390/nu18101511 (PMC13209689; doi:10.3390/nu18101511)
Supplement: Supplementary file 1 [file nutrients-18-01511-s001.zip › nutrients-4287216-supplementary/Supplementary material S4.docx]

**Table 3.** Summary of Study Characteristics and mHealth Intervention Designs

| **Study ID** | **Country** | **Population (N; Intervention/Control; Age; Weight Status)** | **Intervention (mHealth Component)** | **Comparison (Control)** | **Duration** | **Study Design** |
| --- | --- | --- | --- | --- | --- | --- |
| **Johansson et al. (2020) [36]** | Sweden | N=28 (Int: 14; Con: 14); 5–12y; OB | **Provement:** Blinded digital scales + Parent app | UC (Clinic visits) | 6 mo | Pilot RCT |
| **Likhitweerawong et al. (2020) [37]** | Thailand | N=77 (Int: 38; Con: 39); 10–15y; OB (BMI ≥ 95th) | OBEST App: Calorie/weight self-monitoring, goal-setting with rewards, educational tips, and bi-weekly clinician messaging. | Standard obesity care (Thai clinical practice guidelines) | 2 mo | RCT |
| **Delli Bovi et al. (2021) [38]** | Italy | N=103 (Int: 51; Con: 52); 6–14y; OB (BMI >95th) | **PediaFit:** WhatsApp messaging + Hybrid sessions | UC (Standard visits) | 6 mo | RCT |
| **Chai et al. (2021) [39]** | Australia | N=46 (Int: 22; Con: 24); 4–11y; OW/OB risk | **Telehealth:** Web resources + Facebook + SMS | WL | 12 wk | Pilot RCT |
| **Stasinaki et al. (2021) [40]** | Switzerland | N=41 (Int: 21; Con: 20); 10–18y; OB | **PathMate2:** Chatbot + Biofeedback sensors | BCI (Face-to-face visits) | 12 mo | RCT |
| **Vidmar et al. (2022) [41]** | USA | N=117 (AppAlone: 39; AppCoach: 38; Con: 40); 14–18y; OB (BMI >95th) | **Addiction Model:** AppAlone vs. AppCoach | In-person multidisciplinary | 24 wk | 3-arm RCT |
| **Liu et al. (2022) [42]** | China | N=1392 (Int: 691; Con: 701); 8–10y; School-based | **Multifaceted:** School policy + Monitoring App | Usual Practice | 9 mo | Cluster RCT |
| **Salahshoornezhad et al. (2022) [43]** | Iran | N=62 (Int: 31; Con: 31); 9–12y (girls); OW/OB | Game-based nutrition education + CBT | Traditional Lectures | 10 wk | RCT |
| **Memarian et al. (2022) [44]** | Iran | N=46 (Int: 23; Con: 23); 7–11y; OW/OB | **ICT Training:** "Go/No-Go" gamified app | Active Control | 3 mo | RCT |
| **Karssen et al. (2022) [45]** | Netherlands | N=357 (Int: 181; Con: 176); 5–15mo; Infants | **Samen Happie!:** App-based parenting program | WL | 12 mo | RCT |
| **Lee et al. (2023) [46]** | USA | N=73 (Int: 37; Con: 36); 1–3y; Low-income; OB risk | eHealth: Educational videos (based on social cognitive theory), cooking tutorials, and text messages (SMS). | Monthly printed newsletters regarding child nutrition. | 8 weeks | RCT Pilot |
| **Tugault-Lafleur et al. (2023) [32]** | Canada | N=214 (Int: 107; Con: 107); 10–17y; OW/OB | **Aim2Be:** Gamified app + live coach | WL (Brochure) | 3–6 mo | RCT |
| **Alexandrou et al. (2023) [47]** | Sweden | N=552 (Int: 276; Con: 276); 2.5–3y; Preschool | **MINISTOP 2.0:** Multi-language parent app | UC + Brochure | 6 mo | RCT |
| **Tsai et al. (2024) [29]** | Taiwan | N=164 (Int: 82; Con: 82); 6–9y; OW/OB | **FAMILY Program:** Education + monthly SMS support | Active Placebo | 12 mo | RCT |
| **Thorén et al. (2024) [28]** | Sweden | N=65 (Int: 34; Con: 31); 5–12y; OB (IOTF ≥30) | **Web-COP:** Web portal + group sessions | WL (Standard Care) | 6 mo | RCT |
| **Umano et al. (2024) [48]** | Italy | N=75 (Int: 38; Con: 37); 6–12y; OB (BMI ≥95th) | **Nutrilio App:** Parent-led tracking + MD feedback | UC (Lifestyle counseling) | 12 mo | RCT |
| **Davis et al. (2024) [49]** | USA | N=148 (Int: 74; Con: 74); 6–10y; OW/OB (Rural) | **iAmHealthy:** TH (VTC sessions) | Monthly Newsletters | 20 mo | Cluster RCT |
| **Mateo-Orcajada et al. (2024) [50]** | Spain | N=50 (Int: 25; Con: 25); 12–16y; OW/OB | **Strava App:** Step-tracking monitoring | Standard PE classes | 10 wk | RCT |
| **Zhu et al. (2025) [51]** | Australia | N=102 (Int: 51; Con: 51); 7–13y; OW/OB | **BHPO:** Web program + weekly phone coaching | WL | 10 wk | RCT |
| **Foissac et al. (2025) [52]** | France | N=78 (Int: 39; Con: 39); 11–17y; Severe OB | **Remote Monitoring:** App-based self-regulation | Traditional care | 15 mo | RCT |
| **Hagman et al. (2025) [53]** | Sweden | N=428 (Int: 107; Con: 321); 4.0–17.9y; Obesity | **Evira tool: Digitless scale, mobile app, and web clinic interface + physical visits** | Standard pediatric obesity lifestyle care. | 3y | RCT |
| **Wen et al. (2025) [54]** | Australia | N=662 (Int: 331; Con: 331); 2–5y; Prevention | Five nurse-led telephone calls, SMS support, and mailed booklets | Mailed information booklets on unrelated topics (e.g., toilet training) | 2y intervention+ 1 y follow-up | RCT |
| **Audi et al. (2026) [55]** | Brazil | N=60 (Int: 30; Con: 30); 13–17y; OB (ZBMI ≥ +2) | APP Group: FatSecret mobile application for daily dietary self-monitoring + balanced energy-restricted diet | CON Group: Paper-based 3-day food record + balanced energy-restricted diet | 6 months (24 weeks) | RCT (Pilot) |

**Legend:** IOTF: International Obesity Task Force; OB: Obesity; OW: Overweight; WL: Waitlist; UC: Usual Care; TH: Telehealth; VTC: Video-teleconferencing; CBT: Cognitive Behavioral Therapy; BCI: Behavior Changing Intervention; PE: Physical Education; mo: months; wk: weeks; y: years; RCT: Randomized Controlled Trial.

**Table 4.** Clinical Outcomes, Retention & Engagement

| **Study ID** | **Key Clinical Outcomes (Anthropometric/Behavioral)** | **Study Focus** | **Retention Rate** | **mHealth Engagement** |
| --- | --- | --- | --- | --- |
| **Johansson et al. (2020) [36]** | **BMI SDS:** Statistically significant reduction in the Intervention Group (−0.23) compared to Control (+0.01) (p=0.002). | Objective Monitoring | High (89%) | **High:** 80% of participants used digital scales regularly. |
| **Likhitweerawong et al. (2020) [37]** | **BMI**: Sig. reduction in the app group (-0.56 kg/m^2,^ P=0.005); control group sig. increased weight (+1.55 kg, P=0.005). Psychosocial: Sig. improved peer relationship problem scores (P=0.049). | Short-term weight reduction and psychosocial functioning | High: 92% in intervention group (35/38) and 90% in control (35/39). | 31% "good compliance" (≥50% food records sent). |
| **Delli Bovi et al. (2021) [38]** | **zBMI:** Significant reduction in the intensive Intervention Group (p=0.04). **Diet:** Significant increase in fruit/vegetable intake (p=0.02). | Hybrid/Blended Care | Intervention (90%) vs. Control (38%) | **High:** 100% feedback rate on messaging platform. |
| **Chai et al. (2021) [39]** | **Diet:** Significant decrease in energy-dense food intake (p=0.038). **BMI:** No significant difference between groups. | Telehealth + SMS | Good (78%) | **High:** 96% attendance at initial video-teleconferencing sessions. |
| **Stasinaki et al. (2021) [40]** | **BMI-SDS:** No significant difference. **Body Composition:** Significant reduction in body fat percentage in the Intervention Group (p<0.05). | Chatbot & Sensors | Good (94% starters) | **High:** 71.5% daily app usage compliance. |
| **Vidmar et al. (2022) [41]** | **YFAS:** Significant decrease in food addiction symptoms (p=0.045). **BMI:** No significant association with weight loss. | Addiction Model | Low (58%) | **Moderate:** Approximately 11 hours total app usage. |
| **Liu et al. (2022) [42]** | **BMI:** Significant reduction in Intervention vs. Control (Mean Diff: −0.17; p<0.001). Obesity prevalence decreased by 27%. | Multifaceted/School | Excellent (97.8%) | **Dose-response:** Higher app usage correlated with greater weight loss. |
| **Salahshoornezhad et al. (2022) [43]** | **BMI:** Significant reduction in Intervention (−0.55 kg/m²) vs. Control (−0.24 kg/m²) (p=0.01). | Gamified CBT | Excellent (100%) | **High:** 100% compliance with gamified sessions. |
| **Memarian et al. (2022) [44]** | **Diet:** Significant decrease in sweet food intake and choices (p<0.01). **BMI:** No significant anthropometric change. | Inhibitory Control | High (>93%) | **High:** >80% protocol compliance. |
| **Karssen et al. (2022) [45]** | **zBMI:** Significant intervention effect at 6 months (p<0.001); however, the effect was lost/reversed at 12 months. | Infant Prevention | High (88%) | **Low:** Sharp usage decline over time ("App Churn"). |
| **Lee et al. (2023) [46]** | **zBMI**: No significant difference between groups (due to short 8-week duration).  **Nutrition:** Significant increase in fruit (+0.89 servings, p<0.001) and vegetable intake (+0.60 servings, p<0.01). Sedentary: Significant decrease in screen time (-33.87 min, p=0.026). | Early prevention and parental self-efficacy. | 93% | High video view rates and interaction with educational content. |
| **Tugault-Lafleur et al. (2023) [32]** | **zBMI:** No significant difference (p=0.51). **Screen Time:** Significant decrease in Control vs. Intervention (p=0.003). | Gamification & Coach | Low (63%) | **Low:** <50% of participants used the app by Week 2. |
| **De-Jongh González et al. (2022) [33] (Secondary analysis)** | Secondary analysis showed significant decrease only in 'Fully Engaged' parents. | User Engagement Patterns & Digital Phenotypes | Low (63%) | **Low:** <50% of participants used the app by Week 2. |
| **Alexandrou et al. (2023) [47]** | **Diet:** Significant reduction in sweet drink consumption (p<0.001). **BMI:** No significant difference. | Real-world Efficacy | High (93%) | **Moderate:** 54% used the app weekly. |
| **Tsai et al. (2024) [29]** | **zBMI:** Significantly lower in Intervention Group at 12 months (Mean Diff: −0.31; p=0.03). **Body Fat:** Significant reduction (−3.12%). | Family mHealth | Low (53%) | **Passive:** Reliance on SMS and Newsletters. |
| **Thorén et al. (2024) [28]** | **zBMI:** Significant reduction in Intervention Group (−0.27) vs. increase in Control (+0.09) (p<0.001). | Hybrid/Web-COP | High (87%) | **Moderate/High:** 74% parental engagement rate. |
| **Umano et al. (2024) [48]** | **BMI:** No significant additional benefit from app usage alone. **Retention:** App usage significantly reduced attrition rates (p=0.01). | Parent Involvement | High in Intervention (60%) | **Good:** 60% compliance with weekly data entry. |
| **Davis et al. (2024) [49]** | **zBMI:** Significant treatment benefit observed at 20-month follow-up (p=0.048). | Rural Telehealth | High (>87%) | **Moderate:** Completed 15.8h out of 22h recommended treatment dose. |
| **Mateo-Orcajada et al. (2024) [50]** | **Fitness:** Significant within-group increase in muscular endurance. **BMI:** No significant difference. | Passive Tracking | Moderate (83%) | **Critical Failure:** >50% app abandonment after 6 weeks. |
| **Zhu et al. (2025) [51]** | **zBMI:** Significant reduction in Intervention vs. Control (Mean Diff: −0.11; p=0.018). | Web + Phone Coach | Moderate (69%) | **High:** Families completed 9 out of 10 modules. |
| **Foissac et al. (2025) [52]** | **zBMI:** No significant difference between groups (p=0.86). Success Rate: Similar efficacy for weight maintenance (33.3% in App Group vs. 29.7% in Standard Care).  **Success Rate:** Non-inferiority achieved. Remote monitoring was clinically equivalent to face-to-face visits. | Maintenance App | High (92%) | **High:** Effective adolescent autonomy. |
| **Hagman et al. (2025) [53]** | **zBMI:** Adjusted mean change of -0.29 vs. -0.12 in standard care (p=0.02). Remission: 31.8 %vs. 18.7% (p=0.0046). Sustainability: Standard care showed weight regain; digi-physical group sustained loss. | Long-term sustainability of mHealth-integrated care | Higher in intervention: 58 % retention vs. 45 % in standard care at 3 years (p=0.0002). | Home-weighing frequency averaged 2.9 to 3.9 times per week over 3 years. |
| **Wen et al. (2025) [54]** | **BMI:** Significant reduction in mean BMI (diff: -0.30, P=0.039); stronger effect in low-income families (diff: -0.57, P=0.018). Behavioral: Significant improvement in dietary habits (e.g., less TV-side eating) but no effect on play or screen time | Early childhood obesity prevention via telehealth. | 81% at age 3, 74% at age 4, and 61% at age 5 | 58%–64% attended phone sessions; twice-weekly SMS sent (read rates unknown) |
| **Audi et al. (2026) [55]** | **zBMI:** Significant reduction in both groups (APP mean change: -0.28; CON: -0.44; p between groups = 0.543). **Metabolic:** Both groups reduced insulin levels; APP group showed significantly more pronounced improvements in HOMA-IR (P=0.008) and HDL-c (p=0.023). **Behavioral:** Significant reduction in Binge Eating Scale (BES) scores for both groups. | Dietary self-monitoring and metabolic health | Moderate: 60% in APP (18/30) vs. 53.3% in CON (16/30). | Adherence to dietary self-monitoring ranged from 26.7% to 53.3% in the APP group. |

Legend: BMI: Body Mass Index; zBMI: BMI z-score; SDS: Standard Deviation Score; YFAS: Yale Food Addiction Scale; CBT: Cognitive Behavioral Therapy.

**Table 6.** Qualitative Synthesis of Research Gaps, Critical Observations, and Future Directions for Pediatric mHealth Interventions.

| **Study ID** | **Main Finding** | **Research Gap** | **Observations** |
| --- | --- | --- | --- |
| **Johansson et al. (2020) [36]** | Efficacy of Objective Monitoring: Adding a digital support system with daily objective weighing and clinician feedback significantly reduced BMI SDS (-0.24 difference; p=0.002) and improved clinic attendance. | Small Sample Size: As a feasibility trial (N=28), statistical power for secondary outcomes is limited. Long-term sustainability of the "daily weighing" habit remains to be tested in larger cohorts. | Visual Feedback Utility: Parents and clinicians highly valued the visual target curves based on objective data. Unlike self-reported apps, this system integrated real-time medical data, likely enhancing accountability and adherence. |
| **Likhitweerawong et al. (2020) [37]** | The OBEST app significantly reduced BMI and improved peer relationships/prosocial behavior over 2 months. | Needs investigation of long-term outcomes (6–12 months) and factors influencing adherence. Both groups lacked focus on intensive exercise. | Poor compliance remains a challenge; however, even partial app usage showed BMI reduction trends. App features included a photo tool to visualize a thinner self for motivation. |
| **Delli Bovi et al. (2021) [38]** | Hybrid Success: Combining WhatsApp coaching with monthly in-person recall visits significantly reduced BMI z-score (p=0.04) and Screen Time (p=0.04) compared to standard care. | Scalability & Design: Used a "quasi-experimental" design (chronological allocation) and medical students as coaches, raising questions about reproducibility and cost-effectiveness in standard practice. | "Blended Care" Validation: Confirms that mHealth is most effective when used as a complement to frequent human contact (recall visits), rather than a replacement, drastically improving retention (90% vs 38%). |
| **Chai et al. (2021) [39]** | Behavioral Improvement via SMS: A 12-week telehealth intervention did not reduce BMI, but the addition of SMS significantly improved diet quality (less junk food), demonstrating that mobile nudges are effective for behavior change. | Sample Size & Duration: The modest sample size (N=46) and short duration (12 weeks) provided insufficient power to detect changes in weight status (BMI). Authors suggest N=104/group is needed for future efficacy trials. | The "Nudge" Effect: Highlights that low-intensity mHealth tools (SMS) can effectively complement clinical telehealth by keeping parents engaged with nutritional goals between consultations, even in the absence of immediate weight loss. |
| **Stasinaki et al. (2021) [40]** | Human > Digital for BMI: The App group improved body composition (less fat, more muscle) and fitness, but did not lose weight (BMI-SDS) significantly. The Control group (more face-to-face visits) lost significantly more weight initially. | Stress-Weight Link: The study hoped that reducing stress (via biofeedback) would improve weight loss, but found no correlation between cortisol reduction and BMI change. | Chatbot Adherence: The study proves that a Conversational Agent (Chatbot) creates very high adherence (71% daily use), much better than static apps. However, for weight loss, digital tools alone may not be enough compared to intensive human counseling. |
| **Vidmar et al. (2022) [41]** | Correlation Failure: The study demonstrated that while the app helped psychologically (reducing addiction symptoms), this did NOT lead to weight loss. | Weight Disconnect: Clearly shows that a strictly psychological approach ("food addiction") without a strict dietary intervention is insufficient for BMI reduction in adolescents. | COVID Impact: A classic example of a study affected by external factors, but it proves mHealth is a viable alternative for mental health support when physical visits are not possible. |
| **Liu et al. (2022) [42]** | Multifaceted Success: Integrating school-based changes with a parent-focused app significantly reduced BMI and obesity prevalence across socioeconomically distinct regions. | PA Intensity: While behaviors improved, objective moderate-to-vigorous physical activity (MVPA) did not significantly increase, highlighting the challenge of changing exercise intensity. | Parental Monitoring via App: The study proves that mHealth is effective when used as a tool for parents to monitor children (digital surveillance/feedback), creating a strong link between home and school environments. |
| **Salahshoornezhad et al. (2022) [43]** | A multi-disciplinary approach combining mHealth (game), psychology (CBT), and physical activity is significantly more effective than traditional education for weight and metabolic health in the short term. | Short duration (10 weeks) limits conclusions on long-term sustainability. Small sample size of participants. The sample was restricted to girls, limiting generalizability to boys. | This study cannot be included in a zBMI meta-analysis (lack of z-score data). However, it provides strong evidence for the Narrative Synthesis, showing that gamification combined with CBT yields high retention (100%) and metabolic benefits. |
| **Memarian et al. (2022) [44]** | Behavior ≠ Weight Loss: The app successfully trained children to eat fewer sweets (behavior change), but this did not translate to weight loss over 3 months. | Scope of Diet: Targeting only sweets might be insufficient for weight loss if children substitute calories from other sources. A broader focus is likely needed. | Cognitive Training: Demonstrates that gamified cognitive training (Go/No-Go) on a phone is feasible for children (7-11 years) and effective for changing specific eating habits, even if physiological changes (BMI) take longer to appear. |
| **Karssen et al. (2022) [45]** | The app worked short-term (6 mo) for high-risk families (lower education, high parental BMI), but failed long-term (12 mo), potentially due to "Rebound Effect" or loss of engagement. | Low sustained engagement strategies (need for "blended care"). High missing data for zBMI (relied on parent reports). Limited diversity (mostly mothers/Dutch). | Classic example of 'mHealth Fade-out': Short-term efficacy in hard-to-reach groups (Lower SEP) was lost due to poor sustained engagement. Highlights the need for blended care (app + human support) to maintain long-term effects. |
| **Lee et al. (2023) [46]** | The video-based eHealth intervention significantly improved diet quality and reduced sedentary behavior in toddlers. | Small pilot sample size and short duration (8 weeks) limit the ability to detect changes in zBMI; longitudinal studies are needed. | Asynchronous video models are highly accessible for parents in vulnerable groups, effectively overcoming traditional literacy barriers. |
| **Tugault-Lafleur et al. (2023) [32]** | Gamification Trap: The gamified app failed to reduce BMI or improve diet compared to a brochure. Adolescents focused on the "game" and ignored the "lifestyle change" tools. | Engagement Quality: Highlighting that "time spent in app" is a poor metric if users are just playing. Future apps must force interaction with clinical tasks, not just game mechanics. | Waitlist Paradox: Control group improved after access, possibly due to higher readiness. High attrition (37%+) suggests complex apps struggle to retain teens without human accountability. from waiting). The study highlights the limits of complex "self-guided" apps. |
| **Alexandrou et al. (2023) [47]** | Real-world effectiveness trial in primary care showed significant improvements in dietary habits (sweet drinks/treats) and screen time (p<0.05), plus increased Parental Self-Efficacy (PSE), despite no change in zBMI. | Reliance on self-reported behavioral data (risk of social desirability bias). Short duration (6 months) and predominantly normal-weight sample limited the ability to detect BMI changes. | Inclusivity Success: The app was translated (Arabic/Somali /English), successfully reaching 24% foreign-born families. Proves mHealth can be integrated into routine child health care with high retention (93%). |
| **Tsai et al. (2024) [29]** | The app failed to improve weight loss compared to standard care but was highly effective at keeping families engaged in treatment (reducing dropout). | High dropout rates in standard care complicate long-term assessment. Effect on retention faded after the app was removed (at 12 months). | Highlights that mHealth tools act as a "glue" between visits, keeping patients connected to the clinic, even if the app itself doesn't cause extra weight loss. |
| **Thorén et al. (2024) [28]** | Trajectory Mitigation: While "iAmHealthy" did not reduce BMIz significantly post-treatment, it successfully prevented the weight gain observed in the control group over 20 months (p=0.048). | Parental Outcomes & Dose: Parent BMI did not improve (increased in both groups). The actual dose received (15h) was lower than the USPSTF recommendation (26h), limiting efficacy. | Telehealth for Access: Proves that bringing treatment into the home via technology is feasible and maintains high retention in rural populations, acting as a protective factor against the natural trajectory of weight gain. |
| **Umano et al. (2024) [48]** | Inefficacy of Passive Tracking: A 10-week un-gamified walking intervention failed to elicit changes in body composition due to rapid participant disengagement (>50% attrition from app use). | Absence of Gamification: The study utilized a standard tracking tool (Strava) lacking interactive or gamified features. Results suggest that passive self-monitoring is insufficient to sustain motivation in adolescents with obesity. | The "Boredom Factor": Comparison with other studies suggests that without intrinsic motivation or external gamified incentives, adherence to mHealth interventions in this demographic drops precipitously, leading to null clinical outcomes. |
| **Davis et al. (2024) [49]** | Hybrid Success: A web-based program for parents combined with group sessions significantly reduced obesity severity (BMI-SDS) at 6 months, while standard care failed | Component Isolation: Impossible to know if the web program alone would have worked without the group sessions and physical tools (tracker, plate). | Parental Role: Confirms that for younger children (5-12 years), digital interventions must target parents. The control group gained weight, possibly due to the "waitlist effect". |
| **Mateo-Orcajada et al. (2024) [50]** | Sustained Effect: A low-intensity mobile intervention (monthly texts) combined with 3 sessions successfully slowed weight gain (BMIz stabilized) compared to the control group where BMIz increased significantly. | Sleep-Weight Link: Despite being a core component ("SLEEP 9-1"), the intervention failed to improve sleep, suggesting that text messages alone are insufficient for changing sleep habits. | Active Control: The study used a rigorous "Active Placebo" (vegetable education), proving that the weight effects were specific to the lifestyle advice, not just attention from researchers. |
| **Zhu et al. (2025) [51]** | Short-Term Efficacy: A 10-week web-based program supported by weekly phone coaching significantly reduced BMI z-score (mean diff: -0.11) and improved Quality of Life (p=0.034) compared to waitlist control. | Measurement Bias: Anthropometric data (height/weight) were parent-reported using home kits rather than clinically measured, introducing potential bias. Follow-up was limited to 10 weeks. | The Human Element: High adherence (90% module completion) suggests that combining digital content with human accountability (weekly coaching calls) is essential for maintaining engagement in eHealth interventions. |
| **Foissac et al. (2025) [52]** | Cost-Effective Maintenance: A mobile app was non-inferior to traditional face-to-face monitoring for weight loss maintenance (similar success rates ~30%), but significantly reduced the burden of hospital visits and costs. | Age Factor: The intervention was less effective for younger adolescents (<14 years), suggesting that autonomy-based apps require a certain maturity level. | Digital Independence: Unlike many other studies, this app excluded parents to foster adolescent self-regulation. This worked well for older teens but implies a "one size fits all" approach has limits based on age. |
| **Hagman et al. (2025) [53]** | Long-term Superiority: Combining digital tools with physical visits yielded superior 3-year weight loss and remission. | Limited long-term (3y+) mHealth evidence; need for multi-center studies in diverse settings. | Shift from advising to parental empowerment. Daily weighing did not increase the risk of eating disorder diagnoses. |
| **Wen et al. (2025) [54]** | Nurse-led phone/SMS support reduced BMI in preschool children by age 5, especially in low-income households | Limited evidence on telehealth effects during the first 2,000 days of life; lack of cost-effectiveness data. | Telehealth is a viable alternative to face-to-face care during disruptions like COVID-19. Motivational interviewing likely enhanced efficacy. |
| **Audi et al. (2026) [55]** | The FatSecret app and paper records showed equal efficacy for ZBMI reduction. The app group achieved superior metabolic improvements in HOMA-IR and HDL-c. | Strategies are needed to improve low-to-moderate adherence (26.7 %–53.3%). Future research must explicitly evaluate weight-related stigma. | Mobile interfaces may reduce the fear of external judgment/stigma compared to traditional methods. |

Legend: BMI/zBMI: Body Mass Index z-score; SDS: Standard Deviation Score; CBT: Cognitive Behavioral Therapy; PA/MVPA: Physical Activity / Moderate-to-Vigorous Physical Activity; SEP: Socioeconomic Position; USPSTF: United States Preventive Services Task Force; SSB: Sugar-Sweetened Beverages; mHealth Fade-out: The phenomenon of rapid decline in digital engagement after the initial intervention phase; Hybrid Model: An intervention combining mobile technology with human professional support.
